# Ecological Microenvironment Response of Rhizosphere Soil Microbial Communities to Varying Soil Amendments: Insights from Diversity, Stability, and Multi-Functionality

**DOI:** 10.3390/plants15132082

**Published:** 2026-07-03

**Authors:** Yulin Zhang, Junxia Li, Na Qin, Yi Du, Waqar Islam, Sajad Ali, Shutao Dai, Pengyue Li, Cancan Zhu, Chengyang Zhang, Senjie Fu, Ya Jing, Jincang Li, Chunyi Wang

**Affiliations:** 1 Cereal Crop Research Institute, Henan Academy of Agricultural Sciences, Zhengzhou 450002, China; 2 College of Agronomy, Henan Agricultural University, Zhengzhou 450046, China; 3 Institute of Industrial Crops, Henan Academy of Agricultural Sciences, Zhengzhou 450002, China; 4 State Key Laboratory of Desert and Oasis Ecology, State Key Laboratory of Ecological Safety and Sustainable Development in Arid Lands, Xinjiang Institute of Ecology and Geography, Chinese Academy of Sciences, Urumqi 830011, China; 5 Department of Biological Sciences, College of Science, King Faisal University, Al-Ahsa 31982, Saudi Arabia

**Keywords:** continuous cropping obstacles, foxtail millet, rhizosphere soil, soil amendments, multi-functionality

## Abstract

Continuous cropping obstacles (CCOs) severely disrupt the soil physical structure, nutrient cycling, and microbial community balance, leading to decreased crop productivity. However, the effects of soil amendment interventions on bacterial, fungal, and archaeal communities in foxtail millet (*Setaria italica* (L.) P. Beauvois.) systems are not well comprehended. Selected physical, chemical, biological soil amendment and crop rotations were evaluated for their effects on rhizosphere soil microbial diversity, composition, network characteristics, community assembly processes, niche breadth, and multi-functionality. High-throughput sequencing of 16S rRNA and ITS regions demonstrated that earthworm castings significantly enhanced archaeal Chao1, Shannon diversity, and multi-functionality. Meanwhile, *Bacillus mucilaginosus* enhanced fungal diversity, and *B. subtilis* promoted bacterial network complexity. In continuous cropping soil alone, microbial communities exhibited low diversity and were predominantly governed by ecological drift. In contrast, soil amendment treatments shifted assembly toward deterministic processes, including homogeneous and heterogeneous selection. However, the analysis demonstrated greater complexity and niche width in bacterial communities than in fungal or archaeal communities, with keystone modules driven by Actinomycetota, Ascomycota, and Halobacteriota. Structural equation modeling indicated that soil physicochemical properties directly mediated rhizosphere soil microbial alpha diversity, which in turn positively influenced multi-functionality. Overall, organic amendments and microbial inoculants were associated with increases in microbial diversity, network stability, and functionality in this pot experiment, suggesting that such practices may help mitigate CCOs and sustainably improve foxtail millet productivity in dryland agricultural systems.

## 1. Introduction

Continuous cropping obstacles (CCOs) refer to abnormal plant growth resulting from the consecutive cultivation of the same or closely related crop species on the same land, primarily manifested as reduced crop yield and quality decline [1]. Continuous cropping damages soil physical structure, leading to compaction and the degradation of soil aggregates [2], while also inducing chemical imbalances, promoting heavy metal mobilization, and causing persistent nutrient depletion [3,4]. It also disrupts soil microbial community balance, thereby affecting crops and the entire microbial network, triggering various negative effects that lead to CCOs and consequently reduces crop yield and quality [5,6]. Prolonged continuous cropping can shift the soil microbial community towards fungal dominance in a crop- and soil- dependent manner, and has been associated with decreased abundance of functional strains, disrupted nutrient cycling and soil structure, enhanced pathogen infection, increased soilborne diseases, and hindered healthy crop growth and development [1,7].

The rhizosphere is not only a hotspot for microbial interactions but also a critical interface for material exchange between plants and soil [8]. Plant roots release photosynthates as exudates and residues into the soil, supplying carbon and energy to soil microorganisms; in turn, microorganisms convert the organic nutrients into inorganic forms that plants can absorb and use [9]. Continuous cropping cycles diminish beneficial bacterial genera and elevate pathogenic fungal genera in the rhizosphere of watermelon and *Fritillaria pallidiflora*, weakening the root microbial network and decreasing the resilience of the watermelon root-associated microbiome to environmental changes [10,11]. As the duration of continuous cropping on the same plot increases, the abundance of beneficial fungi declines, whereas the types and abundance of harmful fungi increase [12,13]. With more continuous cropping cycles, soil microbial communities transition from being bacteria dominated to fungi dominated, leading to a notable rise in pathogen levels, especially the excessive growth of parasitic Helminthosporium species, which intensifies crop disease occurrence [14,15].

Soil amendment encompasses physical, chemical, and biological methods. Among these, bentonite notably enhances soil labile carbon accumulation, increases both the total quantity and degree of soil humification, enhances soil microbial biomass carbon and nitrogen content, and thereby influences the soil microenvironment and carbon cycling [16,17]. Earthworm castings enhance beneficial bacteria in the soil by boosting the biomass and metabolic activity of rhizosphere microorganisms and suppressing pathogen growth [18,19]. It has been reported to enrich Halobacteriota and increase archaeal diversity and multi-functionality, wherein bacteria exhibit the greatest complexity; fungi show the strongest competitive interactions, and archaea form the simplest yet most tightly connected networks, with Halobacteriota as a core driver [14,20,21]. Biochar increases cation exchange capacity of acidic soils, improves acidic soil aggregation, reduces phenolic acid content through adsorption, and enhances the activity of beneficial soil microorganisms that degrade phenolic acid allelochemicals, thereby alleviating CCOs [22]. Additionally, it promotes the mineralization of soil organic nitrogen, reduces nitrogen leaching, and facilitates nitrogen uptake and utilization by plants [23]. The application of *Bacillus subtilis* and *Trichoderma harzianum*, both individually and in combination, effectively suppressed Phytophthora blight in peppers and enhanced yield [21,24]. Crop rotation and intercropping ameliorate the rhizosphere microenvironment of continuously cropped plants through root exudates, optimize the types and abundance of microorganisms, alleviate continuous cropping damage, and significantly improve photosynthetic efficiency and root vigor in foxtail millet [15,25]. Different cropping systems (continuous maize versus maize and wheat rotation) do not affect soil microbial community structure [26]. Compared with continuous wheat cropping, soybean and wheat rotation reduces soil microbial community richness and Shannon indices [27].

Foxtail millet (*Setaria italica* (L.) P. Beauvois.) is notable for its robust stress resistance, exceptional drought and barrenness tolerance, rich nutritional content (including amino acids, vitamins, carotenoids, and selenium), and high water use efficiency [28,29]. It remains a staple crop in dryland ecological agriculture and plays an indispensable role in food diversity and the structural adjustment of the cropping industry [30,31,32]. Studies have shown that continuous cropping shortens the functional period of foxtail millet leaves, alters soil physicochemical properties and microbial community structure, affects nutrient uptake, and exacerbates the incidence of pests and diseases [15,33]. Currently, research on foxtail millet CCOs has largely focused on changes in soil physicochemical properties, enzyme activities, and crop phenotypic responses [34]. Systematic studies on the composition, phylogenetic relationships, and mechanisms of community assembly in rhizosphere soil microbial communities under different soil amendment treatments remain relatively scarce. In particular, the response patterns of archaeal communities to CCOs have not yet been clearly elucidated. Here, we specifically hypothesize that targeted soil amendments and crop rotation can remodel the composition, diversity, co-occurrence network and multi-functionality of foxtail millet rhizosphere microorganisms, and dominantly shift microbial community assembly from stochastic processes to deterministic processes to alleviate continuous cropping stress. Focusing on this core testable mechanism, this study aimed to: (1) characterize the differences in alpha diversity, beta diversity, network topology and multi-functionality of rhizosphere bacteria, fungi and archaea under different soil amendment and rotation treatments; (2) quantitatively determine the relative contribution of stochastic and deterministic assembly processes of rhizosphere microbial communities via βNTI and Raup–Crick metrics, and clarify how soil amendments regulate the assembly balance of microbial communities; and (3) reveal the successional patterns of three microbial domains under different regulation measures and further clarify the linkage between microbial community assembly processes, plant physiological traits and soil physicochemical properties. This study seeks to provide insight relevant to mitigating CCOs and enhancing foxtail millet cropping systems.

## 2. Results

### 2.1. The Numbers of ASVs, Kingdoms, Phyla, Classes, Orders, Families, Genera, and Species

In the rhizosphere soil (RS) bacterial community, the A13 treatment exhibited a significantly lower number of ASVs compared to A3, A5, A9, and A15 (*p* < 0.05). The number of species was significantly higher in treatments A8 and A9 compared to other treatments (*p* < 0.05) (Figure S1). In the RS fungal community, the A1 treatment resulted in a significantly lower number of ASVs compared to A10 and A13 (*p* < 0.05) (Figure S2). In the RS archaeal community, the A3 treatment exhibited a significantly greater number of ASVs compared to other treatments (*p* < 0.05). The A7 treatment exhibited significantly higher numbers of Kingdoms, Phyla, Classes, Orders, Families, Genera, and Species compared to other treatments, except for A6, A8, A9, and A14 (*p* < 0.05) (Figure S3).

### 2.2. Relative Abundance and Composition of Rhizosphere Soil Microbial Communities

**Figure 1:**
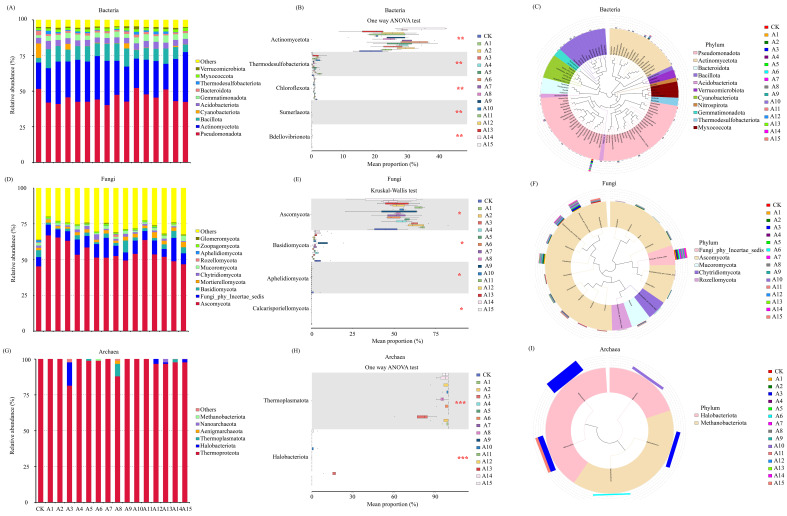
Effects of different soil amendments on bacterial, fungal, and archaeal relative abundance (top 10) and composition of rhizosphere soil. (**A**,**D**,**G**) Relative abundances (top 10) and compositions of rhizosphere soil bacteria, fungi, and archaea. (**B**,**E**,**H**) Phyla with significant differences under different soil amendments. (**C**,**F**,**I**) Linear discriminant effect size (LEfSe) analysis of the bacteria, fungi, and archaea. * *p* < 0.05; ** *p* < 0.01; *** *p* < 0.001. Note: CK, control group; A1, continuous cropping obstacle soil (CCOS); A2, CCOS + corn stalk biochar; A3, CCOS + earthworm castings; A4, CCOS + sodium bentonite; A5, CCOS + fly ash; A6, CCOS + humic acid; A7, CCOS + desulfurization gypsum; A8, CCOS + calcium-magnesium phosphate fertilizer; A9, CCOS + phosphogypsum; A10, CCOS + *Bacillus subtilis*; A11, CCOS + *Bacillus megatherium*; A12, CCOS + seaweed fertilizer; A13, CCOS + *Bacillus mucilaginosus*; A14, Foxtail millet and *Vigna radiata* rotation; A15, Foxtail millet and *Sorghum bicolor* rotation.

For the RS bacterial community, Pseudomonadota and Actinomycetota emerged as the dominant taxa in different treatments, ranging from 40.32% (A7) to 52.34% (A10) and from 17.97% (A13) to 34.95% (A15), respectively (Figure 1A,C; Table S1). Actinomycetota, Thermodesulfobacteriota, Chloroflexota, Sumerlaeota, and Bdellovibrionota showed significant differences in different soil amendment treatments (Figure 1B). For the RS fungal community, Ascomycota emerged as the dominant taxa in different treatments, ranging from 45.35% (CK) to 67.01% (A1) (Figure 1D,F; Table S2). Ascomycota, Basidiomycota, Aphelidiomycota, and Calcarisporiellomycota showed significant differences in different soil amendment treatments (Figure 1E). For the RS archaeal community, Halobacteriota and Thermoplasmatota emerged as the dominant taxa in different treatments (Figure 1G,I; Table S3). Halobacteriota and Thermoplasmatota showed significant differences in different soil amendment treatments (Figure 1H).

### 2.3. Alpha and Beta Diversity of Rhizosphere Soil Microbial Communities

In the RS bacterial community, the Chao1 and Observed features indices were significantly higher in CK, A3, A5, A9, and A15 compared to A13. The Chao1 and Observed features indices for A1 were lower than those for other treatments, except for A13. The CK treatment exhibited a significantly higher Dominance and lower Simpson compared to other treatments. The Shannon in the CK treatment was significantly lower than in all other treatments, except for A9 and A13 (Table 1).

In the RS fungal community, the Chao1 and Observed features indices were notably higher in treatments A10, A12, and A13 than in A1. In treatment A15, the Dominance was significantly higher and the Simpson significantly lower than in other treatments, excluding CK, A6, A7, and A14. The Shannon was notably higher in the A13 treatment compared to CK, A2, A6, and A15, whereas the A15 treatment exhibited significantly lower values than A3, A5, A8, A10, A11, and A13 (Table 2).

In the RS archaeal community, the A3 treatment exhibited significantly higher Chao1, Observed features, and Shannon indices compared to other treatments. The A1 and A6 treatments exhibited a significantly higher Dominance and lower Simpson compared to the A3, A8, and A14 treatments. The Shannon in the A1 treatment was notably lower compared to A3, A8, and A14 (Table 3).

**Figure 2:**
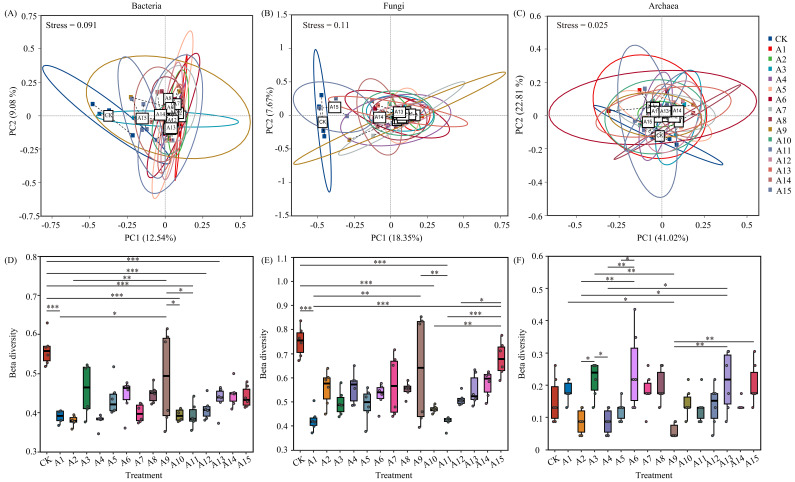
Effects of different soil amendments on bacterial, fungal, and archaeal beta diversity {(**A**–**C**) principal component analysis and (**D**–**F**) Bray–Curtis} of rhizosphere soil. * *p* < 0.05; ** *p* < 0.01; *** *p* < 0.001.

The interpretation rates for RS bacterial, fungal, and archaeal communities were 21.62%, 26.02%, and 63.83%, respectively, and significant differences were also observed in their beta diversity across different soil amendment treatments (Figure 2A–C; Stress < 0.2). The bacterial and fungal beta diversity in the CK treatment was notably greater than in A1, A10, and A11 treatments, whereas the A9 treatment exhibited significantly higher beta diversity compared to A1 and A11 (Figure 2D,E). The fungal beta diversity of the A15 treatment was significantly higher than A1, A10, A11, and A12 (Figure 2E). The archaeal beta diversity was significantly higher in the A3, A6, and A13 treatments compared to A2 and A4 (Figure 2F).

### 2.4. Analysis of Microbial Networks in Rhizosphere Soil

**Figure 3:**
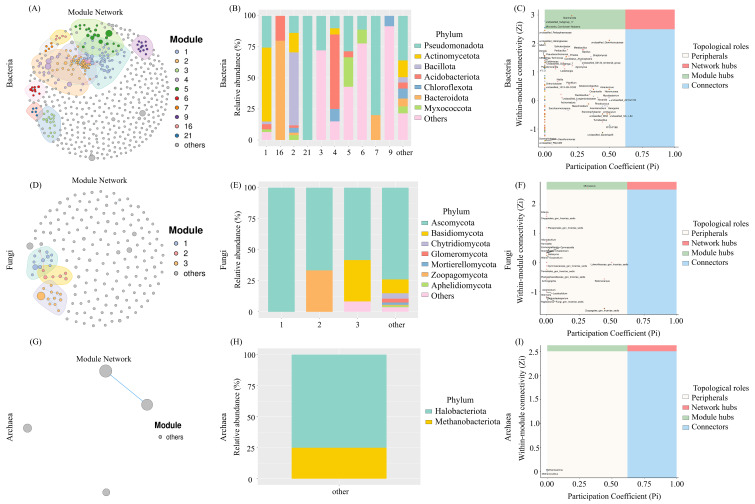
Effects of different soil amendments on bacterial, fungal, and archaeal network characteristics of rhizosphere soil. (**A**,**D**,**G**) Co-occurrence network of rhizosphere soil bacteria, fungi, and archaea. (**B**,**E**,**H**) Relative abundance core phyla of rhizosphere soil bacteria, fungi, and archaea. (**C**,**F**,**I**) These nodes of bacteria, fungi, and archaea were divided into four categories.

The RS microbial community’s structural metrics, including node number, edge number, average path length, average degree, diameter, clustering coefficient, centralized betweenness, and natural connectivity, were observed to be greater in bacteria compared to fungi and archaea (bacteria > fungi > archaea) under various soil amendment treatments (Figure 3A,D,G; Table S4). Archaea exhibited the highest edge density and global efficiency, fungi displayed the highest negative edge proportion, and bacteria showed the highest average weighted degree in the rhizosphere soil microbial community (Table S4). Module 1 of the bacterial microbial community is driven by the phylum level Actinomycetota as the core, module 2 is driven by Bacteroidota as the core, module 4 is driven by Acidobacteriota as the core, modules 7 and 21 are driven by Pseudomonadota as the core, and module 16 is driven by Bacteroidota as the core (Figure 3B). Modules 1, 2, and 3 of the fungal microbial community are driven by the phylum level Ascomycota as the core; however, the module of the archaeal microbial community is driven by Halobacteriota as the core (Figure 3E,H). The bacterial and fungal microbial communities primarily consist of peripherals and module hubs, whereas the archaeal microbial community predominantly comprises peripherals (Figure 3C,F,I).

### 2.5. Community Assembly Process of Rhizosphere Soil Microbial Communities

**Figure 4:**
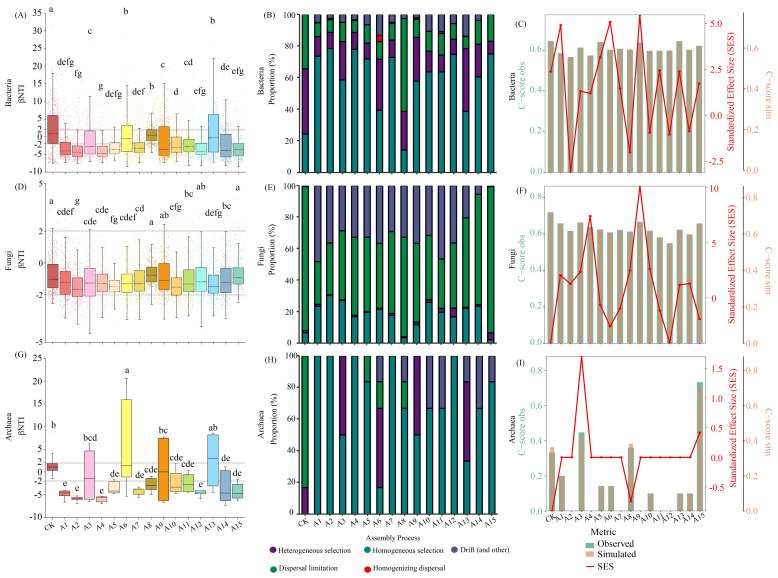
Effects of different soil amendments on bacterial, fungal, and archaeal nearest taxon index (β NTI) (**A**,**D**,**G**) and community assembly process (**B**,**E**,**H**) of rhizosphere soil. (**C**,**F**,**I**) C-score metric of bacteria, fungi, and archaea using null models. Different lowercase letters (a, b, c, d, e, f, and g) indicate that the different soil amendments have significant differences (Duncan’s test, *p* < 0.05).

The β-nearest taxon index of bacterial, fungal, and archaeal communities in the A1 treatment was significantly lower than those in CK and A9 (*p* < 0.05) (Figure 4A,D,G). The bacterial community in the CK treatment was primarily governed by drift, in the A8 treatment was primarily governed by dispersal limitation, in the A13 treatment was primarily governed by heterogeneous selection, whereas in other treatments were influenced by homogeneous selection (Figure 4B). C-score null model analysis indicated that A9 exhibited the highest deterministic process contribution for both bacteria and fungi, whereas A3 did so for archaea (Figure 4C,F,I).

The β-nearest taxon index of fungal communities in the CK, A8, and A15 treatments was significantly higher than in other treatments, except for A9 and A12 (Figure 4D). Fungal communities treated with A1, A2, and A11 were mainly governed by drift, while those in other treatments were influenced by dispersal limitation (Figure 4E). The β-nearest taxon index of archaeal communities in the A6 treatment was significantly higher than in other treatments, except for the A13 treatment (*p* < 0.05) (Figure 4G). The archaeal community in the CK treatment was primarily governed by dispersal limitation, in the A3 and A9 treatments primarily governed by homogeneous selection and heterogeneous selection, in the A13 treatment primarily governed by heterogeneous selection, whereas in the other treatments it was influenced by homogeneous selection (Figure 4H).

### 2.6. Niche Width and Multi-Functionality of Rhizosphere Soil Microbial Communities

**Figure 5:**
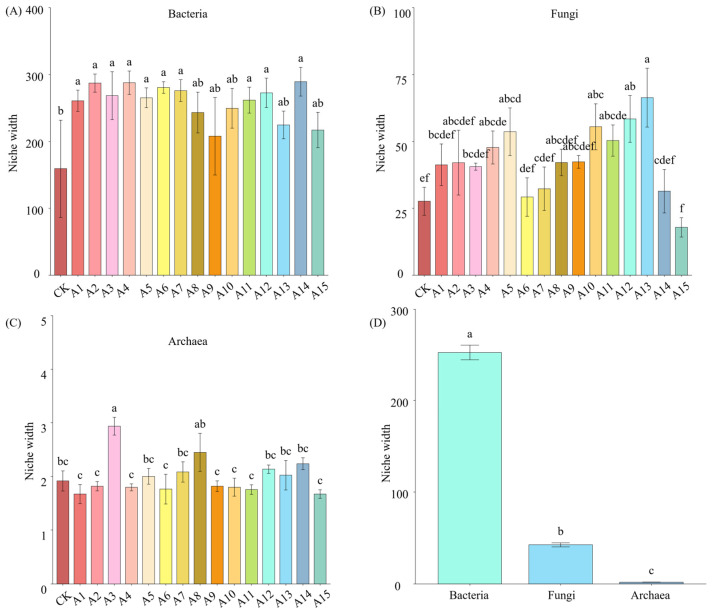
Effects of different soil amendments on rhizosphere soil microbial niche width [(**A**) (bacteria), (**B**) (fungi), (**C**) (archaea) and (**D**) (rhizosphere soi bacterial, fungal, and archaeal niche width)]. Note: CK, control group; A1, continuous cropping obstacle soil (CCOS); A2, CCOS + corn stalk biochar; A3, CCOS + earthworm castings; A4, CCOS + sodium bentonite; A5, CCOS + fly ash; A6, CCOS + humic acid; A7, CCOS + desulfurization gypsum; A8, CCOS + calcium-magnesium phosphate fertilizer; A9, CCOS + phosphogypsum; A10, CCOS + *Bacillus subtilis*; A11, CCOS + *Bacillus megatherium*; A12, CCOS + seaweed fertilizer; A13, CCOS + *Bacillus mucilaginosus*; A14, Foxtail millet and *Vigna radiata* rotation; A15, Foxtail millet and *Sorghum bicolor* rotation. Different lowercase letters (a, b, c, d, e, and f) indicate that the different soil amendments have significant differences (Duncan’s test, *p* < 0.05). The same below.

The bacterial niche width in the CK treatment was notably narrower than in treatments A1, A2, A3, A4, A5, A6, A7, A11, A12, and A14 (Figure 5A; *p* < 0.05). The fungal niche width was significantly higher in the A10, A12, and A13 treatments than in A6 and A15 (Figure 5B; *p* < 0.05). Apart from the A8 treatment, the archaeal niche width was significantly higher in A3 compared to other treatments (Figure 5C; *p* < 0.05). Bacteria exhibited a significantly broader niche width than fungi and archaea, with archaea having the narrowest niche width (Figure 5D; *p* < 0.05).

**Figure 6:**
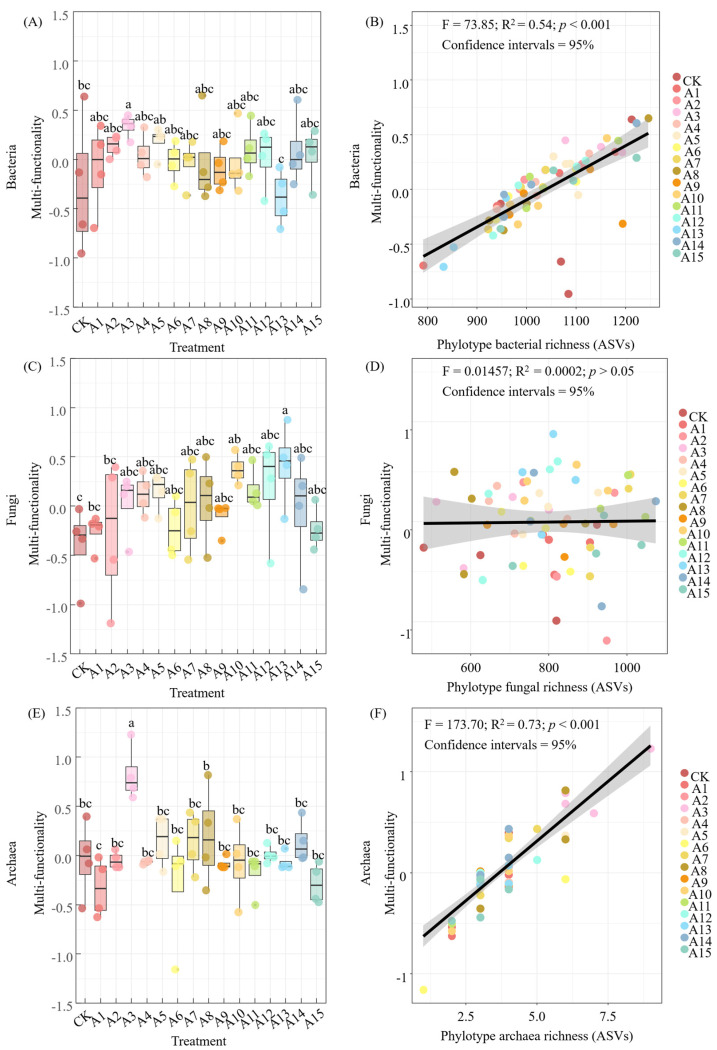
Effects of different soil amendments on rhizosphere soil microbial community multi-functionality. [(**A**,**B**) (rhizosphere soil bacterial multi-functionality)], [(**C**,**D**) (rhizosphere soil fungal multi-functionality)], and [(**E**,**F**) (rhizosphere soil archaeal multi-functionality)]. Different lowercase letters (a, b, and c) indicate that the different soil amendments have significant differences (Duncan’s test, *p* < 0.05).

The bacterial multi-functionality was significantly higher in the A3 treatment than in CK and A13, and markedly lower in the A13 treatment than in A3 and A5 (Figure 6A; *p* < 0.05). The fungal multi-functionality was significantly higher in the A13 treatment than in CK and A1, lower in the A1 treatment than in A13, and higher in the A10 treatment relative to CK (Figure 6C; *p* < 0.05). Archaeal multi-functionality was significantly higher in the A3 treatment than in A1 and A8, and also higher in A3 relative to all other treatments, while it was significantly lower in the A1 treatment than in A8 (Figure 6E; *p* < 0.05). Additionally, as richness increased, the multi-functionality of bacteria and archaea also rose, suggesting enhanced stability (Figure 6B,F; *p* < 0.001). Despite increased richness, fungal multi-functionality did not exhibit a significant upward trend (Figure 6D; *p* > 0.05) (Equations (1) and (2)).

### 2.7. Correlation Analysis: Alpha Diversity, Multi-Functionality, and Plant and Soil Characteristics

**Figure 7:**
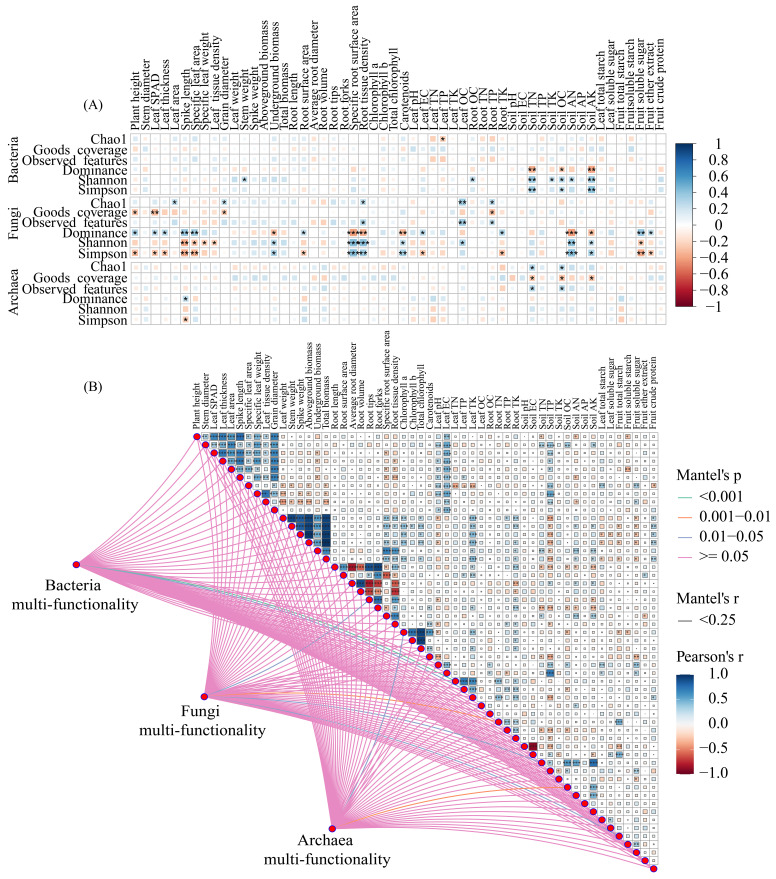
The relationship between rhizosphere soil microbial community alpha diversity [(**A**) bacterial, fungal, and archaeal alpha diversity], multi-functionality [(**B**) bacterial, fungal, and archaeal multi-functionality], and plant and soil traits. Note: Leaf EC, leaf electrical conductivity (μS·cm^−1^); Leaf OC, leaf organic carbon (g·kg^−1^); Leaf TN, leaf total nitrogen (g·kg^−1^); Leaf TP, leaf total phosphorus (g·kg^−1^); Leaf TK, leaf total potassium (g·kg^−1^); Root OC, root organic carbon (g·kg^−1^); Root TN, root total nitrogen (g·kg^−1^); Root TP, root total phosphorus (g·kg^−1^); Root TK, root total potassium (g·kg^−1^); Soil EC, soil electrical conductivity (μS·cm^−1^); Soil OC, soil organic carbon (g·kg^−1^); Soil TN, soil total nitrogen (g·kg^−1^); Soil TP, soil total phosphorus (g·kg^−1^); Soil TK, soil total potassium (g·kg^−1^); Soil AN, soil available nitrogen (mg·kg^−1^); Soil AP, soil available phosphorus (mg·kg^−1^); Soil AK, soil available potassium (mg·kg^−1^). Significance codes: * *p* < 0.05; ** *p* < 0.01; *** *p* < 0.001.

In the RS bacterial community, a significant negative correlation was observed between leaf TP and the bacterial Chao1 (Figure 7A; *p* < 0.05). The bacterial Shannon showed a significant positive correlation with stem weight, root OC, soil TK, and soil AK (Figure 7A; *p* < 0.05).

In the RS fungal community, the Chao1 and Observed features showed a significant positive correlation with root TP; however, Goods coverage showed a significant negative correlation with root TP (Figure 7A; *p* < 0.05). The Dominance showed a significant positive correlation with spike length and specific leaf area. Conversely, it exhibited a notable negative correlation with underground biomass, specific root surface area, root tissue density, carotenoid, soil AN, and soil AK (Figure 7A; *p* < 0.05). The Shannon and Simpson showed an opposite trend compared to the Dominance (Figure 7A; *p* < 0.05).

In the RS archaeal community, soil TN and OC showed a significant negative correlation with the archaeal Goods coverage index, while exhibiting a notable positive correlation with the archaeal Chaol and Observed features indices (Figure 7A; *p* < 0.05). A significant negative correlation was observed between soil AK and the archaeal Goods coverage (Figure 7A; *p* < 0.05). The spike length stem was markedly positively correlated with the archaeal Dominance index, while it was notably negatively correlated with the archaeal Simpson (Figure 7A; *p* < 0.05).

A significant correlation was observed between the bacterial multi-functionality and leaf TN and TP (Figure 7B; *p* < 0.05). The multi-functionality of fungi is significantly correlated with root tips, root TN, soil TN, and fruit total starch (Figure 7B; *p* < 0.05). However, the archaeal multi-functionality exhibited a significant correlation with chlorophyll a and soil OC (Figure 7B; *p* < 0.05).

### 2.8. Path Analysis Diversity, Multi-Functionality, and Plant and Soil Characteristics

**Figure 8:**
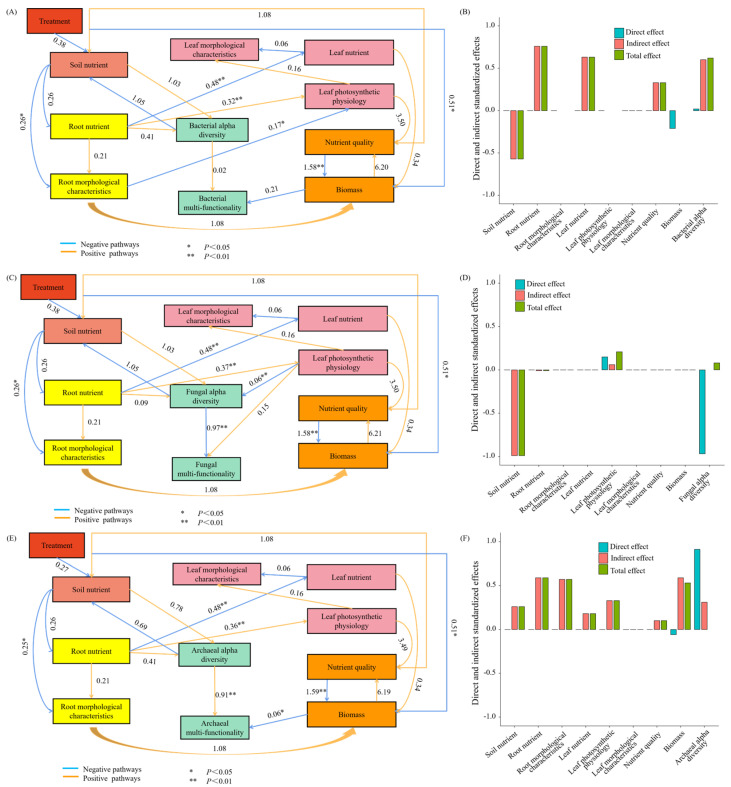
The path analysis among rhizosphere soil microbial multi-functionality [(**A**) (rhizosphere soil bacterial multifunctionality path analysis), (**B**) (rhizosphere soil bacterial multifunctionality direct and indirect standardized effects), (**C**) (rhizosphere soil fungal multifunctionality path analysis), (**D**) (rhizosphere soil fungal multifunctionality direct and indirect standardized effects), (**E**) (rhizosphere soil archaeal multifunctionality path analysis), and (**F**) (rhizosphere soil archaeal multifunctionality direct and indirect standardized effects)], morphological characteristics, and physicochemical properties. Note: (**A**) bacterial multifunctionality model fit indices: n = 64, *X*^2^ = 45.331, *df* = 34, *p* = 0.093, RMSEA = 0.073, GFI = 0.905, CFI = 0.874; (**C**) fungal multifunctionality model fit indices: n = 64, *X*^2^ = 48.019, *df* = 34, *p* = 0.056, RMSEA = 0.081 GFI = 0.894, CFI = 0.933; (**E**) archaeal multifunctionality model fit indices: n = 64, *X*^2^ = 37.65, *df* = 34, *p* = 0.349, RMSEA = 0.073, GFI = 0.916, CFI = 0.989; soil nutrients, including Soil pH, Soil EC, Soil OC, Soil TN, Soil TP, Soil TK, Soil AN, Soil AP, and Soil AK; root nutrients, including Root OC, Root TN, Root TP, and Root TK; root morphological characteristics, including root length, average root diameter, root surface area, root volume, root tips, root forks, specific root surface area and root tissue density; leaf morphological characteristics, including plant height, stem diameter, leaf SPAD, leaf thickness, leaf area, spike length, specific leaf area, specific leaf weight, and leaf tissue density; leaf nutrients, including leaf pH, leaf EC, leaf OC, leaf TN, leaf TP, leaf TK; leaf photosynthetic physiological characteristics, including Chlorophyll a, Chlorophyll b, Total chlorophyll, and Carotenoids; nutrient quality, including fruit crude protein, fruit ether extract, fruit soluble sugar, fruit soluble starch, fruit total starch, leaf soluble sugar, and leaf total starch; biomass, including grain diameter, total biomass, underground biomass, aboveground biomass, spike weight, stem weight, and leaf weight.

The results of the structural equation model (SEM) show that the soil amendment treatments affect root morphological characteristics, root nutrients, leaf photosynthetic physiological characteristics, leaf nutrients, nutrient quality, and biomass by directly influencing the soil physicochemical properties, and indirectly affecting the multi-functionality of rhizosphere soil bacteria, fungi, and archaea (Figure 8A,C,E). For the indirect effects and total effects of rhizosphere soil bacterial multi-functionality, root nutrients had the greatest effect, followed by leaf nutrients, bacterial alpha diversity, and nutrient quality (Figure 8B). The indirect and total effects of leaf photosynthetic physiological characteristics on the rhizosphere soil fungal multi-functionality was greater than soil nutrients, root nutrients, root morphological characteristics, leaf nutrients, leaf morphological characteristics, nutrient quality, biomass, and fungal alpha diversity (Figure 8D). For the indirect effects of rhizosphere soil archaeal multi-functionality, the effect of biomass and root nutrients were the highest (Figure 8F). For the total effect of rhizosphere soil archaeal multi-functionality, the effect of archaeal alpha diversity is the highest (Figure 8F). For the direct effects on rhizosphere soil bacterial and archaeal multi-functionality, the effect of bacterial and archaeal alpha diversity is the highest (Figure 8B,F); however, for the direct effects on rhizosphere soil fungal multi-functionality, the effect of leaf photosynthetic physiological characteristics is the highest (Figure 8D).

## 3. Discussion

### 3.1. Effects of Soil Amendments on the Composition, Diversity, and Functional Characteristics of the Rhizosphere Soil Microbial Community

Prior research indicates that various soil amendments distinctly alter the composition, alpha and beta diversity, network characteristics, community assembly processes, niche breadth, and multi-functionality of rhizosphere soil bacterial, fungal, and archaeal communities in continuous cropping systems [15,19]. Continuous cropping obstacle soil (A1, CCOS) exhibited the highest relative abundance of Ascomycota (67.01%) and the lowest fungal ASVs number. The notable decline in Cyanobacteria from 10.11% in the control group (soil with no crops planted, CK) to 0.06% in A1 (Table S1) further indicates that continuous monocropping selectively eliminates photosynthetic prokaryotes, a hallmark of soil ecological degradation in continuous cropping systems. Fungi, with their extensive hyphal networks and efficient recalcitrant-organic-matter degradation, tend to slow down the turnover of labile nutrients, potentially aggravating nitrogen and phosphorus limitation for crops [35]. The near-elimination of Cyanobacteria—likely driven by reduced light penetration under dense canopies, allelopathic root exudates, or intensified competition for inorganic nitrogen—further diminishes autotrophic carbon input, weakening the soil’s self-regenerative capacity and amplifying disease pressure [36]. These patterns are consistent with the well-documented fungal dominance and microbial diversitydecline under long-term monoculture cropping in medicinal plants such as *Panax notoginseng* and *Strobilanthes sarcorrhiza*, where pathogenic fungi proliferate while beneficial taxa are suppressed [7,37]. The *B. subtilis* (A10) and *B. mucilaginosus* (A13) significantly reconfigured the rhizosphere soil microbial community. The A10 treatment elevated bacterial Pseudomonadota abundance to 52.34%, while A13 enhanced fungal Chao1 and Shannon indices. These responses likely reflect distinct microbial recruitment mechanisms: Pseudomonadota are known for their rapid growth on root-derived labile carbon and their capacity to produce plant-growth-promoting hormones and siderophores, which may explain their enrichment under *B. subtilis*—a well-known biocontrol agent that can alter root exudation patterns [38]. The increase in fungal diversity under *B. mucilaginosus* suggests that this inoculant may modify soil pH or release specific polysaccharides that favor a broader range of saprotrophic fungi, potentially improving decomposition of complex organic matter [21,39]. These findings are consistent with studies on continuous tomato cropping, where farmyard manure combined with *B. subtilis* and *T. harzianum* significantly increased Proteobacteria abundance and enhanced soil enzyme activities, with microbial community functions mainly enriched in amino acid and carbohydrate metabolism pathways [40].

Community assembly analysis showed that A1 consistently exhibited the lowest β-nearest taxon index across rhizosphere soil bacterial, fungal, and archaeal communities, indicating a pronounced shift toward stochastic assembly driven primarily by ecological drift. In contrast, amendments—particularly humic acid (A6), calcium-magnesium phosphate fertilizer (A8), and A13—significantly increased the β-nearest taxon index, indicating enhanced deterministic selection. This finding is consistent with studies on Fritillaria pallidiflora continuous cropping, where prolonged monoculture enhanced stochastic assembly processes and altered microbial functional characteristics [11,41]. According to iCAMP estimates, the direction and magnitude of the stochastic-to-deterministic shift are both amendment-type-specific and microbial domain-dependent, extending previous observations that stochastic processes dominate under certain amendments, whereas deterministic selection prevails under organic fertilizer and microbial inoculants strategies [14,42]. Network analysis revealed domain-dependent structural patterns: bacteria exhibited the greatest network topology with keystone modules driven by Actinomycetota, Bacteroidota, and Pseudomonadota; fungi showed the strongest competitive interactions as reflected by the highest proportion of negative edges; and archaea formed the simplest yet most tightly connected networks with Halobacteriota as the core driver. The higher level of competitive interactions among fungi may reflect intensified niche competition under continuous cropping stress, potentially contributing to the rapid pathogenic shifts documented in monoculture systems [15]. Earthworm castings (A3) significantly improved archaeal Chao1, Shannon indices, and multifunctionality, and uniquely enriched Halobacteriota (16.30% versus 0% in most treatments). This is ecologically significant because archaea, though often overlooked, play critical roles in ammonia oxidation and organic-matter turnover under stress conditions. The selective stimulation of Halobacteriota by earthworm casts may be mediated by the release of specific organic acids or by improving soil aeration and moisture, conditions that favor these oligotrophic archaea [43]. This aligns with evidence that organic fertilizers, particularly vermicompost-derived products, significantly promote the abundance and diversity of rhizospheric archaea, and that earthworm castings serve as organic soil amendments, positively impacting soil biological properties and promoting beneficial microorganisms [44,45]. A beneficial microbial (*Pseudomonas* sp. RU47, *B. atrophaeus* ABi03, *T. harzianum* OMG16) consortium promoted maize growth and drought resilience across farming systems by enhancing iron uptake, hormonal balance, and detoxification, while simultaneously increasing microbial network complexity [21]. Collectively, these results imply that amendment-driven restructuring is not a uniform phenomenon; rather, each amendment exerts a domain-specific selective pressure that alters key functional guilds.

### 3.2. Relationship Between the Rhizosphere Soil Microbial Community and Plant Growth, Physiology, Morphology, Nutrient Content, as Well as Soil Physicochemical Properties

Correlation analyses identified domain-specific linkages among rhizosphere microbial diversity, plant traits, and soil properties. For bacteria, soil total nitrogen, organic carbon, and available potassium were positively correlated with Shannon and Simpson indices, while leaf total phosphorus was negatively correlated with the Chao1 index. These patterns are consistent with findings in the jujube orchard, where biochar combined with bentonite significantly increased the bacterial Shannon index, with soil organic matter driving microbial functional shifts via urease activity pathways [46]. Similarly, in continuous tomato systems, organic amendments enriched Proteobacteria and Bacteroidota, with soil hydrolyzable nitrogen, available phosphorus, and pH identified as key environmental drivers of community restructuring [8]. For fungi, the Chao1 was positively correlated with leaf area, grain diameter, root tissue density, leaf organic carbon, and root total phosphorus, linking fungal diversity to plant photosynthetic capacity and phosphorus uptake. The Dominance exhibited trends opposite to those of the Simpson of fungi, revealing a trade-off wherein higher fungal Dominance was associated with enhanced aboveground vegetative and reproductive development, whereas higher Simpson (reflecting greater community evenness) was linked to increased root elaboration, carotenoid accumulation, and soil nutrient availability [47,48,49].

For the rhizosphere soil archaeal community, soil total nitrogen and organic carbon were positively correlated with Chao1 and Observed features indices, while spike length was positively correlated with archaeal Dominance index, suggesting reproductive organ development associates with less even archaeal communities. The bacterial multi-functionality is significantly associated with leaf nitrogen and phosphorus, fungal multi-functionality with root tips, root total nitrogen, soil total nitrogen, and fruit total starch, and archaeal multi-functionality with chlorophyll a and soil organic carbon. The bacterial and archaeal multi-functionality exhibited significant positive relationships with species richness, whereas fungal multi-functionality did not. This domain-dependent pattern is consistent with findings that biochar-amended continuous cropping soils reduced fungal diversity while increasing bacterial diversity, and that key taxa such as Monographella, Acremonium, and Geosmithia enhanced nutrient availability and suppressed Fusarium, indicating that fungal functional outcomes are driven more by compositional turnover than by richness per se [36]. In soil microcosm studies, organic carbon amendments—including humic acid and plant-derived compounds—have been shown to stimulate distinct archaeal subpopulations and significantly alter community composition [50], while broader niche breadths in common bacterial taxa are typically associated with stronger deterministic selection and greater functional contributions [51]. Furthermore, organic fertilizers can shift archaeal abundance and composition by modifying soil properties such as available phosphorus and potassium [52], supporting the interpretation that organic amendments create unique microhabitats capable of fostering archaeal diversification and decoupling richness—functional relationships in a domain-dependent manner [14,53]. Structural equation modeling revealed that soil amendments directly influenced soil properties, mediating rhizosphere soil microbial alpha diversity and ultimately affecting multi-functionality. For rhizosphere soil bacteria and archaea, alpha diversity exerted the highest direct effect on multi-functionality, whereas for fungi, leaf photosynthetic physiological characteristics had the highest direct effect. This domain-dependent regulation is consistent with evidence that bacterial multi-functionality was more governed by diversity, while fungal multi-functionality was more coupled to plant physiological signals through root exudation [54,55], highlighting the need for integrated management approaches targeting both soil properties and plant traits to restore ecosystem functionality in continuous cropping systems.

### 3.3. Limitations and Future Directions

The structural equation model has limited statistical power due to 64 observations and 34 degrees of freedom, so path coefficients and latent variable structures need further validation with larger datasets. The microbial co-occurrence networks were constructed based on Spearman’s correlation of ASV abundance. Such correlation-based networks only reflect synchronous or asynchronous abundance shifts among taxa, and cannot directly represent real ecological interactions such as mutualism, competition, antagonism or cross-feeding relationships. This pot experiment was conducted under controlled conditions, and factors such as the historical planting legacy of the tested soil, soil water-holding capacity, seasonal environmental fluctuations, and fine-scale soil heterogeneity were not explicitly incorporated into the analysis. In addition, all test soils were collected from a single continuous cropping field in the Huang-Huai-Hai plain, which may restrict the applicability of conclusions to other soil types or climatic zones.

Future research can be expanded from the following aspects. The structural equation model should be further validated using large-sample field survey data to improve model reliability. Culture-dependent co-culture assays, metagenomic functional profiling and gene expression quantification should be adopted to verify the actual ecological functions and interspecific interactions of core microbial taxa, moving beyond correlation-based inference to mechanistic interpretation. Finally, multi-site long-term field trials across diverse soil types and agroclimatic zones are needed, incorporating variables such as soil historical legacy, hydrological properties and seasonal dynamics. Meanwhile, combined application schemes of different soil amendments should be explored to develop more efficient and widely applicable strategies for alleviating foxtail millet continuous cropping obstacles.

## 4. Materials and Methods

### 4.1. Study Location and Botanical Specimens

The experiment was conducted from July to November 2025 at the Henan research and development base for modern agriculture in Xinxiang, Henan province, China (113°42′9.252″ E, 35°0′31.2588″ N). The region experiences an average temperature range from 10 °C (low) to 22 °C (high), with extremes reaching 41 °C (high) and −12 °C (low) and receives an annual rainfall of 832.6 mm. Seeds of the foxtail millet cultivar “Zhenggu 678” were used as the pot experiment material. The soils used in the experiment were collected from fields subjected to 5 years of continuous foxtail millet cropping, as well as from fields under 5-year foxtail millet–*Vigna radiata* and foxtail millet–*Sorghum bicolor* rotation systems. Foxtail millet has a fibrous root system without a distinct taproot, characterized by dense, highly branched roots whose depth and distribution dynamically vary with soil texture (deeper in sandy soil, shallower in clay) and field management, with over 80% of the root system concentrated in the 0–40 cm surface layer and the highest density occurring in the 0–20 cm layer [56]. Thus, soil samples were collected from a depth of 0–30 cm, then sieved and air-dried. Based on the dosage provided by the manufacturer and referring to the dosages in other experiments, the application rates per pot (0.075 m^2^) were as follows: corn stalk biochar, 60 g [57]; earthworm castings, 375 g; sodium bentonite, 10 g [57]; fly ash, 300 g; humic acid, 3.375 g [58]; desulfurization gypsum, 600 g; phosphogypsum, 135 g; calcium-magnesium phosphate fertilizer, 60 g; *B. subtilis*, 4.5 g [59]; *B. megaterium*, 0.5 g; *B. mucilaginosus*, 0.5 g; seaweed fertilizer, 4.5 g. The soil conditioner was purchased from Wang Miao Nong Ye Ke Ji Co., Ltd. (Zhengzhou, China). The soil was not subjected to pre-incubation prior to planting, thus maintaining its original field state. Firstly, the air-dried soil was placed into plastic flower pots that were 35 cm in height and 30 cm in diameter. Each pot contained 10 kg of soil. A tray was placed under each pot to allow the water that leaks out during watering to be absorbed and reused later. Secondly, 12 different amendments were added to each of the 12 plastic flower pots at once, and they were mixed evenly using a gardening shovel. The field capacity of the soil in the pots was measured to be about 20%, and water was replenished by weighing to maintain the soil moisture content at 75% to 80% of the field capacity. Meanwhile, humic acid and microbial inoculants were applied as a spray treatment 1 week after seedling emergence. All other management measures were consistent with local field management methods.

### 4.2. Experimental Designs

The experiment was conducted on 1 July 2025. For the whole growth period, to each pot was added once 0.07 g·kg^−1^ urea and 0.03 g·kg^−1^ potassium phosphate. Twenty seeds were planted in each pot and watered daily to keep soil moisture at 75–80% of field capacity. Foxtail millet seedlings were thinned 10 days post-sowing to promote uniform growth. In each pot, only 10 seedlings were maintained. Sixteen treatments were established as follows: (CK) Control group (soil with no crops planted), (A1) continuous cropping obstacle soil (CCOS), (A2) CCOS + corn stalk biochar, (A3) CCOS + earthworm castings, (A4) CCOS + sodium bentonite, (A5) CCOS + fly ash, (A6) CCOS + humic acid, (A7) CCOS + desulfurization gypsum, (A8) CCOS + calcium-magnesium phosphate fertilizer, (A9) CCOS + phosphogypsum, (A10) CCOS + *B. subtilis*, (A11) CCOS + *B. megatherium*, (A12) CCOS + seaweed fertilizer, (A13) CCOS + *B. mucilaginosus*, (A14) Foxtail millet and *V. radiata* rotation, and (A15) Foxtail millet and *S. bicolor* rotation. Using a randomized block experimental design, each treatment included four pots, constituting four replications, totaling 64 pots. Every three days, the pots were rearranged to equalize their exposure to the surrounding environment.

### 4.3. Determination of Plant Traits

(1) At the stage of maximum biomass, plant height and spike length of foxtail millet were measured using a tape measure, and stem diameter, spike diameter, and leaf thickness were determined with a vernier caliper (CD-15AXW, Mitutoyo, Inc., Tokyo, Japan). Leaf SPAD values were recorded using a handheld chlorophyll meter (SPAD-502, Konica minolta sensing, Inc., Tokyo, Japan). Healthy leaves collected from the mid-to-upper portion of the stem were divided into two portions: one portion was used for leaf area determination, and the other was utilized for the quantification of chlorophyll a, chlorophyll b, and carotenoid contents. Leaf area was measured using ImageJ version 1.52t software (Softonic International, Barcelona, Spain) according to the standard procedure. Subsequently, leaves were transferred to an oven set at 75 °C for 24 h to determine LDW. Specific leaf area was calculated as leaf area divided by LDW, and specific leaf weight was calculated as LDW divided by leaf area. Foxtail millet spikes were first collected using scissors, after which all aboveground parts were harvested by cutting at the stem base with pruning shears and transported to the laboratory. Leaves and stems were separated, cut into small pieces, and then transferred to an oven at 75 °C for 24 h before biomass determination. A precision electronic balance (FA1004, manufactured in Tianjin, China) with a readability of 0.001 g was used to measure grain thousand weight, spike weight, grain weight, leaf biomass, stem biomass, and root biomass. The original data are shown in Tables S10 and S12.
(2) At the stage of maximum biomass, foxtail millet root systems were collected using the water washing method. Roots were carefully spread out in a root tray containing a small amount of deionized water, and intertwined roots were gently separated using forceps to avoid compromising scanning quality. A root scanner (J221A, Seiko Epson Corp., Suwa, Japan) was employed to capture root images, and root system architecture was subsequently analyzed using root analysis software (HED-WinRHIZO, v2016) to determine the total root length, average root diameter, root surface area, root volume, root tips, and root forks. After scanning, roots were oven-dried at 75 °C to a constant weight, and the root dry weight was recorded. The original data is shown in Table S11. Specific root surface area and root tissue density were calculated using the following formulas:
  Specific root surface area (cm^2^·g^−1^) = Root surface area/Root dry weight
  Root tissue density (g·cm^−3^) = Root dry weight/(Root surface area × Root thickness)

### 4.4. Determination of Plant and Soil Nutrient Indicators

The ether extract contents of leaves and fruits were determined according to the Chinese national standard GB/T 6433-2006 [60]. Soluble sugar and soluble starch contents were quantified using the anthrone–sulfuric acid colorimetric method. The crude protein content was measured by the Kjeldahl method. Standard methods were used to measure the Root OC, Leaf OC, Soil OC, TN, TP, TK, AN, AP, and AK as well as leaf and soil pH and EC [61]. The K_2_Cr_2_O_7_–H_2_SO_4_ oxidation method was utilized to determine Root OC, Leaf OC, and Soil OC, and a Kjeldahl Nitrogen Analyzer (K1160, Jinan Hanon Instruments Co., Ltd., Jinan, China) was employed to measure the TN concentration. AN was measured through the alkali hydrolysis method, and TP and TK were determined using an Inductively Coupled Plasma-Optical Emission Spectrometer (iCAP 6300, Thermo Elemental, Waltham, MA, USA) after the samples were digested in concentrated HNO_3_. A colorimetric analysis was performed to extract AP using HCl/NH_4_F and ascorbic acid molybdate on a continuous flow autoanalyzer (Autoanalyzer 3, Bran and Luebbe, Norderstedt, Germany). The NH_4_OAc extraction method was employed to determine AK. The leaf and soil pH were determined using a pH meter (PHSJ-6 L, INESA Scientific Instrument Co., Ltd., Shanghai, China) at a soil/water ratio of 1:2.5 (*w*/*v*), and the leaf and soil EC were measured at a soil/water ratio of 1:5 (*w*/*v*) using an EC meter (DDSJ-319 L, INESA Scientific Instrument Co., Ltd.). The original data are shown in Tables S13–S16.

### 4.5. Collecting Samples of Root-Associated Microorganisms and Conducting Measurements

The rhizosphere soil (RS) samples were collected from plant species at depths of 0 to 25 cm. A vortex oscillator brush was used to collect RS closely associated with fine roots (≤2 mm) by collecting soil from the roots. The collected soil was transferred into individual sterile centrifuge tubes. All samples were immediately stored at −80 °C in an ultra-low temperature freezer to preserve microbial integrity before DNA extraction.

Genomic DNA was extracted from 0.5 g of RS using a Qiagen DNA extraction kit (Venlo, The Netherlands). PCR amplification focused on the bacterial and archaeal 16S rRNA gene V3–V4 region using primers 341F (5′-CCTAYGGGRBGCASCAG-3′) and 806R (5′-GGACTACNNGGGTATCTAAT-3′), as well as the fungal ITS 1–5 F region with primers 5-1737F (5′-GGAAGTAAAAGTCGTAACAAGG-3′) and 2-2043R (5′-GCTGCGTTCTTCATCGATGC-3′). Each sample was demultiplexed from the sequencing data using its barcode and PCR primer sequences. After removing barcode and primer sequences, the reads for each sample were merged using FLASH (v1.2.11) [62] to generate raw tag sequences. The raw tags were processed with FASTP (v0.23.1) using strict quality-filtering parameters to produce high-quality clean tags [63]. Chimeric sequences were identified and removed using VSEARCH (v2.16.0) by aligning tags to a reference species annotation database to enhance sequence quality. The resulting high-quality, chimera-free sequences were retained as effective tags for downstream analysis [64]. Further sequence filtering was conducted utilizing QIIME II software (v202202) [65]. The DADA2 plugin was employed for quality control, denoising, splicing, and chimera removal to produce amplicon sequence variants (ASVs) [66]. Bacterial and archaeal ASVs were identified using the RDP classifier at a 70% confidence threshold, the Mothur method, and the SSUrRNA database from Silva version 138.1 with a threshold range of 0.8–1 [67,68]. Fungal ASVs were identified using the UNITE database [69]. The data preprocessing statistics and quality control are shown in Tables S8 and S9.

### 4.6. Assessing Multi-Functionality

The alpha diversity’s original data are shown in Tables S5–S7. To obtain a quantitative multi-functionality of the RS (bacteria, fungi, and archaea) of foxtail millet from the different soil amendments, complementary multi-functionality approaches were used: the average multi-functionality approach provides an easy-to-interpret and straight forward measure of the ability of an ecosystem to simultaneously sustain multiple functions, and this approach is widely used by current multi-functionality studies [70,71]. The following calculation methods were used:

Six α-diversity indices closely related to species diversity (Chao1, Observed features, Dominance, Goods coverage, Simpson, and Shannon) were selected to represent multi-functionality. First of all, these α-diversity indices were normalized, and then their Z-scores were calculated. The calculation formula is as follows:

(1) $$Z=\left(x_{i}-\lambda_{i}\right)/\delta_{i}$$

where Z is the Z-score of the α-diversity index, and x_i_ is the value of the α-diversity index parameter; *λ*_i_ is the average value of the α-diversity index. *δ*_i_ is the standard deviation of the α-diversity index. Averaging the Z-score of α-diversity is called average multi-functionality. The calculation formula is:

(2) EMF=∑i6xi6

### 4.7. Statistics Analysis

All statistical analyses and plots were performed using R (v4.5.3) [72]. Data normality was assessed using the Shapiro–Wilk test, and the Kruskal–Wallis test was applied when the normality assumption was violated. The alpha diversity (Chao1, Observed features, Dominance, Goods coverage, Simpson, and Shannon index) at the ASVs level was calculated using QIIME2. For the calculation of beta diversity, the vectorized ASVs matrix based on the Bray–Curtis distance was adopted. To evaluate the influence of different soil amendments on alpha and beta diversity, a one-way analysis of variance (ANOVA) and *t*-test were performed, respectively. Differences between means were assessed using Duncan’s test, with a significance level of *p* < 0.05. Principal component analysis (PC) was employed to analyze variations in bacterial, fungal, and archaeal communities (based on ASV levels) using vegan (v2.7-5) package (using PERMANOVA with 999 permutations). The Spaa (v0.2.5) package (method = Levins) was employed to calculate the niche width of bacteria, fungi, and archaea in the RS, with visualization achieved through the ggplot2 (v4.0.0) package [73]. The Mantel test with 999 permutations was employed to assess Spearman’s correlations between the multi-functionality of bacteria, fungi, and archaea and various factors, including leaf nutrient, leaf photosynthetic physiological characteristics, leaf morphological characteristics, total biomass, root morphological characteristics, root nutrients, plant nutrient quality, and soil nutrients using the corrplot (v0.9.5), linkET (v0.1.0), and microeco (v2.0.0) packages.

Co-occurrence networks were constructed based on the Spearman’s correlation (absolute correlation coefficient > 0.7 and FDR-adjusted *p* < 0.001) matrix among ASVs in Gephi (v0.92) (accessed on 15 April 2026). Additionally, the topological roles of nodes were determined according to the connectivity between nodes within modules (Zi) and between modules (Pi) using the igraph (v2.2.2) and ggClusterNet (v2.0.0) packages. These nodes were divided into four categories: peripherals (those that have few connections to other nodes within and between modules, Zi < 2.5 and Pi < 0.62), connectors (nodes that link the modules, Pi > 0.62), module hubs (nodes that generate more connections within the module, Zi > 2.5), and network hubs (nodes that are highly connected throughout the network, Zi > 2.5 and Pi > 0.62) [74].

These processes were quantified using the phylogenetic bin-based null model framework iCAMP (v1.5.12) [75], which extends the null model [76] and partitions assembly into five processes: homogeneous selection, heterogeneous selection, dispersal limitation, homogeneous dispersal, and drift. Community clustering or overdispersion was evaluated by comparing observed metrics with null model expectations using the checkerboard score (C-score) [77]. ASV tables were converted to binary presence–absence matrices, and standardized effect sizes were calculated as the deviation of observed values from the null model mean divided by the null model standard deviation [78,79]. C-scores were assessed using 30,000 simulations and the sequential swap algorithm implemented in EcoSimR (v0.1.0).

Firstly, the first axis of leaf nutrient, leaf photosynthetic physiological characteristics, leaf morphological characteristics, biomass, root nutrients, root morphological characteristics, nutrient quality, soil nutrients, and RS alpha diversity (bacteria, fungi, and archaea) were selected through principal component analysis. Second, the structural equation model (SEM) implemented in IBM SPSS Amos 24 software (IBM Corp., Armonk, NY, USA, 2016) was utilized to investigate the causal relationships among leaf nutrient, leaf photosynthetic physiological characteristics, leaf morphological characteristics, biomass, root nutrients, root morphological characteristics, nutrient quality, soil nutrients, RS alpha diversity (bacteria, fungi, and archaea), and RS multi-functionality (bacteria, fungi, and archaea).

## 5. Conclusions

Continuously cropping soil alone produced low-diversity communities governed by ecological drift, whereas soils following amendment soils imposed deterministic selection and improved alpha and beta diversity. Bacteria enabled the most complex networks, fungi exhibited the strongest competitive interactions, and archaea developed the simplest yet most tightly connected networks. Bacterial and archaeal multi-functionality were directly governed by alpha diversity, whereas fungal multi-functionality was primarily driven by leaf photosynthetic physiological traits. Archaea exhibited the narrowest niche width; however, the module of the archaeal microbial community was driven by Halobacteriota as the core. Through this pot experiment, the best soil amendment was selected. In the future, different soil amendments can be mixed and treated, and verification experiments can be conducted in the field to select better combinations to mitigate CCOs.

## Figures and Tables

**Table 1:** Effects of different soil amendments on bacterial alpha diversity of rhizosphere soil.

	Treatment	Chao1	Observed Features	Dominance	Goods Coverage	Simpson	Shannon
Bacteria	CK	1093.21 ± 59.28 a	1067.75 ± 54.70 a	0.018 ± 0.0094 a	0.996 ± 0.001 a	0.982 ± 0.0094 b	8.45 ± 0.39 b
	A1	1015.56 ± 92.56 ab	994.00 ± 83.43 ab	0.004 ± 0.0003 b	0.997 ± 0.001 a	0.996 ± 0.0003 a	8.94 ± 0.09 a
	A2	1070.02 ± 41.73 ab	1050.50 ± 34.70 ab	0.003 ± 0.0003 b	0.997 ± 0.001 a	0.997 ± 0.0003 a	9.05 ± 0.04 a
	A3	1137.09 ± 41.44 a	1114.50 ± 35.07 a	0.004 ± 0.0008 b	0.996 ± 0.001 a	0.996 ± 0.0008 a	9.12 ± 0.04 a
	A4	1044.87 ± 45.42 ab	1025.75 ± 38.37 ab	0.004 ± 0.0003 b	0.997 ± 0.001 a	0.997 ± 0.0003 a	9.03 ± 0.05 a
	A5	1107.90 ± 22.76 a	1081.25 ± 10.45 a	0.004 ± 0.0003 b	0.996 ± 0.001 a	0.996 ± 0.0003 a	9.03 ± 0.03 a
	A6	1051.79 ± 50.64 ab	1024.75 ± 43.48 ab	0.004 ± 0.0003 b	0.996 ± 0.001 a	0.997 ± 0.0003 a	9.01 ± 0.04 a
	A7	1016.49 ± 26.96 ab	995.25 ± 26.88 ab	0.004 ± 0.0003 b	0.997 ± 0.0004 a	0.996 ± 0.0003 a	8.98 ± 0.06 a
	A8	1041.48 ± 68.23 ab	1022.50 ± 65.40 ab	0.004 ± 0.0005 b	0.997 ± 0.001 a	0.996 ± 0.0005 a	8.93 ± 0.11 a
	A9	1095.18 ± 59.71 a	1064.00 ± 49.85 a	0.009 ± 0.0056 b	0.995 ± 0.001 a	0.991 ± 0.0056 a	8.79 ± 0.19 ab
	A10	1059.01 ± 48.18 ab	1033.25 ± 44.56 ab	0.004 ± 0.0004 b	0.996 ± 0.001 a	0.996 ± 0.0004 a	8.94 ± 0.10 a
	A11	1086.27 ± 47.98 ab	1056.00 ± 43.66 ab	0.004 ± 0.0003 b	0.996 ± 0.001 a	0.996 ± 0.0003 a	9.02 ± 0.07 a
	A12	1056.14 ± 53.64 ab	1033.75 ± 45.39 ab	0.004 ± 0.0005 b	0.996 ± 0.001 a	0.997 ± 0.0005 a	8.99 ± 0.09 a
	A13	906.30 ± 34.62 b	898.25 ± 32.76 b	0.005 ± 0.0005 b	0.998 ± 0.0004 a	0.995 ± 0.0005 a	8.79 ± 0.09 ab
	A14	1052.78 ± 68.92 ab	1034.75 ± 63.75 ab	0.003 ± 0.0003 b	0.997 ± 0.001 a	0.997 ± 0.0003 a	9.06 ± 0.09 a
	A15	1111.19 ± 62.29 a	1079.25 ± 56.81 a	0.005 ± 0.0005 b	0.995 ± 0.001 a	0.995 ± 0.0005 a	8.94 ± 0.04 a

Note: CK, control group; A1, continuous cropping obstacle soil (CCOS); A2, CCOS + corn stalk biochar; A3, CCOS + earthworm castings; A4, CCOS + sodium bentonite; A5, CCOS + fly ash; A6, CCOS + humic acid; A7, CCOS + desulfurization gypsum; A8, CCOS + calcium-magnesium phosphate fertilizer; A9, CCOS + phosphogypsum; A10, CCOS + *Bacillus subtilis*; A11, CCOS + *Bacillus megatherium*; A12, CCOS + seaweed fertilizer; A13, CCOS + *Bacillus mucilaginosus*; A14, Foxtail millet and *Vigna radiata* rotation; A15, Foxtail millet and *Sorghum bicolor* rotation. Different lowercase letters (a and b) indicate that the different soil amendments have significant differences (Duncan’s test, *p* < 0.05). The occurrence of a standard deviation of 0 is because all four sets of data are identical. Therefore, we have included this set of original data in the attached materials.

**Table 2:** Effects of different soil amendments on fungal alpha diversity of rhizosphere soil.

	Treatment	Chao1	Observed Features	Dominance	Goods Coverage	Simpson	Shannon
Fungi	CK	729.51 ± 82.43 ab	700.25 ± 74.80 bc	0.043 ± 0.012 ab	0.999 ± 0.00065 a	0.957 ± 0.012 ab	6.31 ± 0.39 cd
	A1	645.78 ± 27.42 b	640.75 ± 27.12 c	0.027 ± 0.005 b	1.000 ± 0.00029 a	0.973 ± 0.005 a	6.70 ± 0.17 abcd
	A2	729.48 ± 89.85 ab	712.00 ± 89.74 abc	0.034 ± 0.014 b	0.999 ± 0.00025 a	0.966 ± 0.014 a	6.57 ± 0.52 bcd
	A3	769.79 ± 64.89 ab	756.25 ± 67.06 abc	0.025 ± 0.001 b	0.999 ± 0.00000 a	0.975 ± 0.001 a	7.04 ± 0.04 abc
	A4	836.07 ± 52.38 ab	818.25 ± 45.40 abc	0.022 ± 0.003 b	0.999 ± 0.00025 a	0.978 ± 0.003 a	7.00 ± 0.12 abc
	A5	831.86 ± 9.39 ab	812.25 ± 9.68 abc	0.021 ± 0.004 b	0.999 ± 0.00025 a	0.980 ± 0.004 a	7.15 ± 0.17 abc
	A6	799.46 ± 66.28 ab	763.50 ± 54.70 abc	0.041 ± 0.009 ab	0.998 ± 0.00048 a	0.959 ± 0.009 ab	6.54 ± 0.15 bcd
	A7	887.88 ± 16.89 a	854.00 ± 29.48 abc	0.043 ± 0.017 ab	0.998 ± 0.00058 a	0.957 ± 0.017 ab	6.75 ± 0.29 abcd
	A8	859.35 ± 71.96 ab	824.50 ± 68.67 abc	0.025 ± 0.003 b	0.998 ± 0.00048 a	0.975 ± 0.003 a	6.97 ± 0.22 abc
	A9	790.58 ± 28.06 ab	762.50 ± 19.52 abc	0.024 ± 0.001 b	0.999 ± 0.00029 a	0.976 ± 0.001 a	6.82 ± 0.11 abcd
	A10	932.09 ± 82.35 a	903.50 ± 72.12 ab	0.020 ± 0.004 b	0.999 ± 0.00065 a	0.980 ± 0.004 a	7.31 ± 0.12 ab
	A11	868.50 ± 49.39 ab	847.75 ± 46.32 abc	0.021 ± 0.002 b	0.999 ± 0.00029 a	0.979 ± 0.002 a	7.07 ± 0.14 abc
	A12	895.32 ± 129.41 a	864.00 ± 120.95 ab	0.019 ± 0.003 b	0.998 ± 0.00063 a	0.982 ± 0.003 a	7.21 ± 0.31 ab
	A13	950.61 ± 80.54 a	926.00 ± 76.22 a	0.017 ± 0.003 b	0.998 ± 0.00048 a	0.984 ± 0.003 a	7.44 ± 0.20 a
	A14	868.01 ± 94.65 ab	835.00 ± 84.83 abc	0.044 ± 0.016 ab	0.998 ± 0.00048 a	0.956 ± 0.016 ab	6.61 ± 0.38 abcd
	A15	831.59 ± 67.50 ab	808.25 ± 58.11 abc	0.062 ± 0.009 a	0.999 ± 0.00050 a	0.939 ± 0.009 b	6.04 ± 0.19 d

Note: CK, control group; A1, continuous cropping obstacle soil (CCOS); A2, CCOS + corn stalk biochar; A3, CCOS + earthworm castings; A4, CCOS + sodium bentonite; A5, CCOS + fly ash; A6, CCOS + humic acid; A7, CCOS + desulfurization gypsum; A8, CCOS + calcium-magnesium phosphate fertilizer; A9, CCOS + phosphogypsum; A10, CCOS + *Bacillus subtilis*; A11, CCOS + *Bacillus megatherium*; A12, CCOS + seaweed fertilizer; A13, CCOS + *Bacillus mucilaginosus*; A14, Foxtail millet and *Vigna radiata* rotation; A15, Foxtail millet and *Sorghum bicolor* rotation. Different lowercase letters (a, b, c, and d) indicate that the different soil amendments have significant differences (Duncan’s test, *p* < 0.05). The occurrence of a standard deviation of 0 is because all four sets of data are identical. Therefore, we have included this set of original data in the attached materials.

**Table 3:** Effects of different soil amendments on archaeal alpha diversity of rhizosphere soil.

	Treatment	Chao1	Observed Features	Dominance	Goods Coverage	Simpson	Shannon
Archaea	CK	3.25 ± 0.48 b	3.25 ± 0.48 bc	0.532 ± 0.053 ab	0.99 ± 0.01 ab	0.47 ± 0.05 bc	1.20 ± 0.17 bcd
	A1	3.00 ± 0.71 b	2.75 ± 0.48 c	0.64 ± 0.06 a	0.98 ± 0.02 ab	0.36 ± 0.06 c	0.91 ± 0.16 d
	A2	3.25 ± 0.25 b	3.25 ± 0.25 bc	0.54 ± 0.02 ab	0.99 ± 0.01 ab	0.46 ± 0.02 bc	1.18 ± 0.07 bcd
	A3	6.96 ± 1.07 a	6.25 ± 0.63 a	0.35 ± 0.02 c	0.90 ± 0.03 c	0.65 ± 0.02 a	1.99 ± 0.10 a
	A4	3.50 ± 0.50 b	3.25 ± 0.25 bc	0.54 ± 0.02 ab	0.98 ± 0.02 ab	0.46 ± 0.02 bc	1.19 ± 0.01 bcd
	A5	4.00 ± 0.71 b	3.75 ± 0.48 bc	0.50 ± 0.05 abc	0.98 ± 0.02 ab	0.50 ± 0.05 abc	1.35 ± 0.14 bcd
	A6	2.75 ± 0.63 b	2.75 ± 0.63 c	0.64 ± 0.12 a	0.99 ± 0.01 ab	0.36 ± 0.12 c	0.94 ± 0.33 cd
	A7	3.50 ± 0.29 b	3.50 ± 0.29 bc	0.50 ± 0.07 abc	1.00 ± 0.00 a	0.50 ± 0.07 abc	1.33 ± 0.18 bcd
	A8	4.50 ± 0.89 b	4.25 ± 0.75 b	0.45 ± 0.06 bc	0.95 ± 0.02 b	0.55 ± 0.06 ab	1.49 ± 0.23 b
	A9	3.00 ± 0.001 b	3.00 ± 0.001 bc	0.55 ± 0.03 ab	1.00 ± 0.00 a	0.45 ± 0.03 bc	1.14 ± 0.07 bcd
	A10	3.25 ± 0.48 b	3.25 ± 0.48 bc	0.58 ± 0.06 ab	0.99 ± 0.01 ab	0.42 ± 0.06 bc	1.12 ± 0.18 bcd
	A11	2.75 ± 0.25 b	2.75 ± 0.25 c	0.56 ± 0.02 ab	1.00 ± 0.00 a	0.44 ± 0.02 bc	1.07 ± 0.08 bcd
	A12	3.25 ± 0.25 b	3.25 ± 0.25 bc	0.48 ± 0.02 abc	0.99 ± 0.01 ab	0.52 ± 0.02 abc	1.30 ± 0.06 bcd
	A13	3.00 ± 0.001 b	3.00 ± 0.001 bc	0.53 ± 0.05 ab	1.00 ± 0.00 a	0.47 ± 0.05 bc	1.18 ± 0.10 bcd
	A14	3.50 ± 0.29 b	3.50 ± 0.29 bc	0.45 ± 0.02 bc	0.99 ± 0.01 ab	0.55 ± 0.02 ab	1.43 ± 0.10 bc
	A15	2.75 ± 0.25 b	2.75 ± 0.25 c	0.61 ± 0.04 ab	0.99 ± 0.01 ab	0.39 ± 0.04 bc	0.96 ± 0.08 cd

Note: CK, control group; A1, continuous cropping obstacle soil (CCOS); A2, CCOS + corn stalk biochar; A3, CCOS + earthworm castings; A4, CCOS + sodium bentonite; A5, CCOS + fly ash; A6, CCOS + humic acid; A7, CCOS + desulfurization gypsum; A8, CCOS + calcium-magnesium phosphate fertilizer; A9, CCOS + phosphogypsum; A10, CCOS + *Bacillus subtilis*; A11, CCOS + *Bacillus megatherium*; A12, CCOS + seaweed fertilizer; A13, CCOS + *Bacillus mucilaginosus*; A14, Foxtail millet and *Vigna radiata* rotation; A15, Foxtail millet and *Sorghum bicolor* rotation. Different lowercase letters (a, b, c, and d) indicate that the different soil amendments have significant differences (Duncan’s test, *p* < 0.05). The occurrence of a standard deviation of 0 is because all four sets of data are identical. Therefore, we have included this set of original data in the attached materials.

## Data Availability

The raw sequencing data are deposited in the National Microbiology Data Center (NMDC) (https://nmdc.cn/resource/genomics/sra; (accessed on 25 May 2026)) with accession numbers https://nmdc.cn/resource/genomics/sra/detail/NMDC40124853-NMDC40124920; (accessed on 25 May 2026) (bacteria) and https://nmdc.cn/resource/genomics/sra/detail/NMDC40124921-NMDC40124988; (accessed on 25 May 2026) (fungi). Bacterial data are deposited in the National Microbiology Data Center (NMDC) with accession numbers NMDC40124853, NMDC40124854, NMDC40124855, NMDC40124856, NMDC40124857, NMDC40124858, NMDC40124859, NMDC40124860, NMDC40124861, NMDC40124862, NMDC40124863, NMDC40124864, NMDC40124865, NMDC40124866, NMDC40124867, NMDC40124868, NMDC40124869, NMDC40124870, NMDC40124871, NMDC40124872, NMDC40124873, NMDC40124874, NMDC40124875, NMDC40124876, NMDC40124877, NMDC40124878, NMDC40124879, NMDC40124880, NMDC40124881, NMDC40124882, NMDC40124883, NMDC40124884, NMDC40124885, NMDC40124886, NMDC40124887, NMDC40124888, NMDC40124889, NMDC40124890, NMDC40124891, NMDC40124892, NMDC40124893, NMDC40124894, NMDC40124895, NMDC40124896, NMDC40124897, NMDC40124898, NMDC40124899, NMDC40124900, NMDC40124901, NMDC40124902, NMDC40124903, NMDC40124904, NMDC40124905, NMDC40124906, NMDC40124907, NMDC40124908, NMDC40124909, NMDC40124910, NMDC40124911, NMDC40124912, NMDC40124913, NMDC40124914, NMDC40124915, NMDC40124916, NMDC40124917, NMDC40124918, NMDC40124919, NMDC40124920; Fungal data are deposited in the National Microbiology Data Center (NMDC) with accession numbers NMDC40124921, NMDC40124922, NMDC40124923, NMDC40124924, NMDC40124925, NMDC40124926, NMDC40124927, NMDC40124928, NMDC40124929, NMDC40124930, NMDC40124931, NMDC40124932, NMDC40124933, NMDC40124934, NMDC40124935, NMDC40124936, NMDC40124937, NMDC40124938, NMDC40124939, NMDC40124940, NMDC40124941, NMDC40124942, NMDC40124943, NMDC40124944, NMDC40124945, NMDC40124946, NMDC40124947, NMDC40124948, NMDC40124949, NMDC40124950, NMDC40124951, NMDC40124952, NMDC40124953, NMDC40124954, NMDC40124955, NMDC40124956, NMDC40124957, NMDC40124958, NMDC40124959, NMDC40124960, NMDC40124961, NMDC40124962, NMDC40124963, NMDC40124964, NMDC40124965, NMDC40124966, NMDC40124967, NMDC40124968, NMDC40124969, NMDC40124970, NMDC40124971, NMDC40124972, NMDC40124973, NMDC40124974, NMDC40124975, NMDC40124976, NMDC40124977, NMDC40124978, NMDC40124979, NMDC40124980, NMDC40124981, NMDC40124982, NMDC40124983, NMDC40124984, NMDC40124985, NMDC40124986, NMDC40124987, NMDC40124988. The paper includes the study’s original data. The data on the physical and chemical properties of plants and soil have been used in prior articles. In this article, only a correlation analysis has been conducted. For additional questions, please contact the first or corresponding author.

## Supplementary Materials

The following supporting information can be downloaded at: https://www.mdpi.com/article/10.3390/plants15132082/s1, Figure S1: Effects of different soil amendments on the number of bacterial ASVs, Kingdom, Phylum, Class, Order, Family, Genus, and Species; Figure S2: Effects of different soil amendments on the number of fungal ASVs, Kingdom, Phylum, Class, Order, Family, Genus, and Species; Figure S3: Effects of different soil amendments on the number of archaeal ASVs, Kingdom, Phylum, Class, Order, Family, Genus, and Species; Table S1: The relative abundances of dominant bacteria (top 10 phyla) of rhizosphere soil among different soil amendment treatments; Table S2: The relative abundances of dominant fungi (top 10 phyla) of rhizosphere soil among different soil amendment treatments; Table S3: The relative abundances of dominant archaea (top 10 phyla) of rhizosphere soil among different soil amendment treatments; Table S4: Network co-occurrence properties of rhizosphere soil among different soil amendment treatments; Table S5: Effects of different soil amendments on bacterial alpha diversity; Table S6: Effects of different soil amendments on fungal alpha diversity; Table S7: Effects of different soil amendments on archaeal alpha diversity; Table S8: Data preprocessing statistics and quality control of rhizosphere soil bacteria; Table S9: Data preprocessing statistics and quality control of rhizosphere soil fungi; Table S10: Effects of different soil amendments on leaf traits; Table S11: Effects of different soil amendments on root traits; Table S12: Effects of different soil amendments on leaf photosynthetic pigment content, crop biomass, and yield; Table S13: Effects of different soil amendments on nutritional quality of leaves and fruits; Table S14: Effects of different soil amendments on leaf physicochemical properties; Table S15: Effects of different soil amendments on root nutrients; Table S16: Effects of various soil amendments on the soil physicochemical properties.

## Abbreviations

The following abbreviations are used in this manuscript:

CCOs	Continuous cropping obstacles
CCOS	Continuous cropping obstacle soil
RS	Rhizosphere soil
LDW	Leaf dry weight
SPAD	Soil and plant analyzer development
Leaf EC	Leaf electrical conductivity
TN	Total nitrogen
TP	Total phosphorus
TK	Total potassium
AN	Available nitrogen
AP	Available phosphorus
AK	Available potassium
EC	Electrical conductivity
Leaf OC	Leaf organic carbon
Leaf TN	Leaf total nitrogen
Leaf TP	Leaf total phosphorus
Leaf TK	Leaf total potassium
Root OC	Root organic carbon
Root TN	Root total nitrogen
Root TP	Root total phosphorus
Root TK	Root total potassium
Soil EC	Soil electrical conductivity
Soil OC	Soil organic carbon
Soil TN	Soil total nitrogen
Soil TP	Soil total phosphorus
Soil TK	Soil total potassium
Soil AN	Soil available nitrogen
Soil AP	Soil available phosphorus
Soil AK	Soil available potassium
