# Supplementary material for: Ecological Microenvironment Response of Rhizosphere Soil Microbial Communities to Varying Soil Amendments: Insights from Diversity, Stability, and Multi-Functionality
Source: Plants (Basel). 2026 Jul 3;15(13):2082. doi: 10.3390/plants15132082 (PMC13364074; doi:10.3390/plants15132082)
Supplement: Supplementary file 1 [file plants-15-02082-s001.zip › plants-4371573-supplementary.pdf]

Supplementary material

Ecological microenvironment response of rhizosphere soil microbial communities to varying soil amendments: Insights from diversity, stability, and multi-functionality

Yulin Zhang[1,†], Junxia Li[1,2,*,†], Na Qin[1,2], Yi Du[3], Waqar Islam[4], Sajad Ali[5], Shutao Dai[1], Pengyue Li[1], Cancan Zhu[1], Chengyang Zhang[1], Senjie Fu[1], Ya Jing[1], Jincang Li[1] and Chunyi Wang[1]

[1] Cereal Crop Research Institute, Henan Academy of Agricultural Sciences, Zhengzhou 450002, China

[2] College of Agronomy, Henan Agricultural University, Zhengzhou 450046, China

[3] Institute of Industrial Crops, Henan Academy of Agricultural Sciences, Zhengzhou 450002, China

[4] State Key Laboratory of Desert and Oasis Ecology, State Key Laboratory of Ecological Safety and Sustainable Development in Arid Lands, Xinjiang Institute of Ecology and Geography, Chinese Academy of Sciences, Urumqi 830011, China

[5] Department of Biological Sciences, College of Science, King Faisal University, Al-Ahsa 31982, Saudi Arabia.

*Address correspondence to: lijunxia@126.com

[†]These authors contributed equally to this work.

Table S1. The relative abundance of dominant bacteria (top 10 phyla) of rhizosphere soil among different soil amendment treatments

	Treatment	Pseudomonadota	Actinomycetota	Bacillota	Cyanobacteriota	Acidobacteriota	Gemmatimonadota	Bacteroidota	Thermodesulfobacteriota	Myxococcota	Verrucomicrobiota	Others
Bacteria	CK	51.69%	18.48%	3.26%	10.11%	3.97%	1.85%	3.10%	0.40%	1.45%	0.82%	4.87%
	A1	42.09%	24.11%	13.39%	0.06%	5.72%	3.39%	1.36%	2.38%	1.62%	0.95%	4.93%
	A2	41.06%	29.77%	10.89%	0.08%	4.18%	3.53%	1.08%	1.02%	1.78%	1.18%	5.43%
	A3	45.75%	25.12%	8.36%	4.04%	3.98%	3.74%	1.38%	0.63%	1.44%	0.87%	4.69%
	A4	42.62%	29.46%	9.61%	0.06%	3.97%	4.05%	1.00%	1.19%	1.80%	0.77%	5.47%
	A5	42.66%	28.19%	10.79%	0.13%	4.28%	3.02%	1.69%	0.98%	1.74%	1.37%	5.15%
	A6	44.24%	30.47%	8.94%	0.41%	3.70%	3.47%	1.36%	0.54%	1.39%	0.94%	4.55%
	A7	40.32%	31.04%	11.89%	0.24%	3.94%	3.27%	1.36%	0.14%	1.95%	0.74%	5.11%
	A8	47.53%	23.84%	12.20%	0.25%	4.04%	4.05%	1.03%	0.77%	1.30%	0.56%	4.42%
	A9	42.77%	24.57%	12.70%	3.58%	3.86%	2.78%	1.75%	0.59%	1.70%	1.33%	4.38%
	A10	52.34%	20.58%	6.03%	0.19%	4.53%	4.20%	1.98%	1.90%	1.78%	2.08%	4.40%
	A11	47.86%	24.29%	8.16%	0.30%	4.70%	4.76%	1.64%	1.05%	1.55%	0.74%	4.96%
	A12	45.55%	25.66%	8.61%	0.05%	4.86%	3.97%	1.30%	1.90%	2.00%	1.19%	4.91%
	A13	51.30%	17.97%	10.72%	0.03%	4.10%	5.12%	1.07%	1.64%	1.74%	1.17%	5.15%
	A14	43.11%	29.54%	8.40%	0.81%	4.19%	3.96%	1.22%	0.25%	1.98%	1.03%	5.52%
	A15	42.55%	34.95%	5.30%	0.17%	3.80%	4.07%	1.36%	0.08%	2.25%	0.57%	4.90%

Note: CK, control group; A1, Continuous cropping obstacle soil (CCOS); A2, CCOS + corn stalk biochar; A3, CCOS + earthworm castings; A4, CCOS + sodium bentonite; A5, CCOS + fly ash; A6, CCOS + humic acid; A7, CCOS + desulfurization gypsum; A8, CCOS + calcium-magnesium-phosphate fertilizer; A9, CCOS + phosphogypsum; A10, CCOS + *Bacillus subtilis*; A11, CCOS + *Bacillus megatherium*; A12, CCOS + seaweed fertilizer; A13, CCOS + *Bacillus mucilaginosus*; A14, Foxtail millet and *Vigna radiata* rotation; A15, Foxtail millet and *Sorghum bicolor* rotation.

Table S2. The relative abundance of dominant fungi (top 10 phyla) of rhizosphere soil among different soil amendment treatments

	Treatment	Ascomycota	Fungi_phy_Incertae_sedis	Basidiomycota	Mortierellomycota	Chytridiomycota	Mucoromycota	Rozellomycota	Aphelidiomycota	Zoopagomycota	Glomeromycota	Others
Fungi	CK	45.35%	6.47%	4.85%	1.69%	1.53%	1.93%	0.40%	1.36%	0.06%	0.35%	36.00%
	A1	67.01%	7.44%	1.46%	2.19%	0.23%	0.32%	0.05%	0.14%	1.13%	0.26%	19.78%
	A2	65.53%	5.68%	0.74%	1.47%	0.75%	1.40%	0.03%	0.10%	1.24%	0.19%	22.85%
	A3	63.23%	6.71%	1.75%	1.29%	1.03%	0.71%	0.06%	0.05%	0.84%	0.31%	24.01%
	A4	53.48%	10.65%	3.00%	2.08%	1.95%	2.26%	0.14%	0.13%	0.84%	0.20%	25.26%
	A5	58.56%	8.04%	2.36%	2.31%	2.32%	0.73%	0.11%	0.13%	0.89%	0.53%	24.01%
	A6	51.48%	9.45%	1.29%	1.77%	3.78%	0.86%	0.04%	0.06%	0.81%	0.14%	30.32%
	A7	51.43%	13.88%	3.03%	2.34%	0.88%	2.06%	0.03%	0.18%	1.35%	0.59%	24.23%
	A8	52.56%	7.17%	2.01%	1.62%	1.25%	1.34%	1.72%	0.31%	0.90%	0.35%	30.77%
	A9	49.46%	5.66%	8.29%	2.68%	2.01%	2.02%	0.02%	0.09%	0.83%	0.30%	28.64%
	A10	54.09%	9.29%	2.21%	2.55%	1.13%	0.92%	0.25%	0.19%	1.13%	0.23%	28.00%
	A11	63.78%	6.29%	1.98%	1.78%	1.25%	1.01%	0.15%	0.15%	1.10%	0.16%	22.35%
	A12	53.71%	9.73%	1.74%	4.08%	1.18%	1.72%	0.13%	0.30%	1.10%	0.41%	25.89%
	A13	52.02%	5.49%	3.23%	2.56%	1.45%	1.85%	0.24%	0.12%	0.89%	0.42%	31.74%
	A14	48.93%	16.29%	4.90%	2.43%	0.86%	1.07%	0.22%	0.02%	0.95%	0.44%	23.89%
	A15	46.81%	7.68%	4.21%	3.69%	0.64%	3.62%	0.06%	0.05%	0.39%	0.46%	32.38%

Note: CK, control group; A1, Continuous cropping obstacle soil (CCOS); A2, CCOS + corn stalk biochar; A3, CCOS + earthworm castings; A4, CCOS + sodium bentonite; A5, CCOS + fly ash; A6, CCOS + humic acid; A7, CCOS + desulfurization gypsum; A8, CCOS + calcium-magnesium-phosphate fertilizer; A9, CCOS + phosphogypsum; A10, CCOS + *Bacillus subtilis*; A11, CCOS + *Bacillus megatherium*; A12, CCOS + seaweed fertilizer; A13, CCOS + *Bacillus mucilaginosus*; A14, Foxtail millet and *Vigna radiata* rotation; A15, Foxtail millet and *Sorghum bicolor* rotation.

Table S3. The relative abundance of dominant archaea (top 10 phyla) of rhizosphere soil among different soil amendment treatments

	Treatment	Thermoproteota	Halobacteriota	Thermoplasmatota	Aenigmarchaeota	Nanoarchaeota	Methanobacteriota	Others
Archaea	CK	100.00%	0.00%	0.00%	0.00%	0.00%	0.00%	0.00%
	A1	100.00%	0.00%	0.00%	0.00%	0.00%	0.00%	0.00%
	A2	100.00%	0.00%	0.00%	0.00%	0.00%	0.00%	0.00%
	A3	81.52%	16.30%	0.00%	0.00%	0.00%	0.00%	2.17%
	A4	100.00%	0.00%	0.00%	0.00%	0.00%	0.00%	0.00%
	A5	98.91%	0.00%	1.09%	0.00%	0.00%	0.00%	0.00%
	A6	98.91%	0.00%	0.00%	0.00%	0.00%	1.09%	0.00%
	A7	100.00%	0.00%	0.00%	0.00%	0.00%	0.00%	0.00%
	A8	88.04%	0.00%	8.70%	2.17%	0.00%	0.00%	1.09%
	A9	100.00%	0.00%	0.00%	0.00%	0.00%	0.00%	0.00%
	A10	100.00%	0.00%	0.00%	0.00%	0.00%	0.00%	0.00%
	A11	100.00%	0.00%	0.00%	0.00%	0.00%	0.00%	0.00%
	A12	96.74%	3.26%	0.00%	0.00%	0.00%	0.00%	0.00%
	A13	96.74%	1.09%	0.00%	0.00%	2.17%	0.00%	0.00%
	A14	97.83%	0.00%	2.17%	0.00%	0.00%	0.00%	0.00%
	A15	97.83%	2.17%	0.00%	0.00%	0.00%	0.00%	0.00%

Note: CK, control group; A1, Continuous cropping obstacle soil (CCOS); A2, CCOS + corn stalk biochar; A3, CCOS + earthworm castings; A4, CCOS + sodium bentonite; A5, CCOS + fly ash; A6, CCOS + humic acid; A7, CCOS + desulfurization gypsum; A8, CCOS + calcium-magnesium-phosphate fertilizer; A9, CCOS + phosphogypsum; A10, CCOS + *Bacillus subtilis*; A11, CCOS + *Bacillus megatherium*; A12, CCOS + seaweed fertilizer; A13, CCOS + *Bacillus mucilaginosus*; A14, Foxtail millet and *Vigna radiata* rotation; A15, Foxtail millet and *Sorghum bicolor* rotation.

Table S4. Network co-occurrence properties of rhizosphere soil among different soil amendment treatments

	Node number	Edge number	Edge density	Negative_percentage	Average path length	Global efficiency	Average degree	Average weighted degree	Diameter	Clustering coefficient	Centralized betweenness	Natural connectivity
Bacteria	519	637	0.0047	0.03	4.56	0.0245	2.45	0.83	15	0.52	0.0163	13.48
Fungi	190	50	0.0028	0.04	2.75	0.0066	0.53	0.17	7	0.43	0.0062	0.73
Archaea	4	1	0.1667	0	1.00	0.1667	0.50	0.18	1	0	0	0.24

Note: (1) Node number: the total number of nodes (vertices) in the network. (2) Edge number: the total number of edges (links) in the network. (3) Edge density: the ratio of the actual number of edges to the maximum possible number of edges in the network. (4) Negative_percentage: the proportion of negative edges relative to the total number of edges in the network. (5) Average path length: the average of the shortest path distances between all pairs of nodes. It measures the overall efficiency of information flow in the network. (6) Global efficiency: the average of the reciprocals of the shortest path distances between all pairs of nodes. It measures how efficiently information is exchanged across the network. (7) Average degree: the average of the degrees of all nodes. It reflects the overall connectivity density of the network. (8) Average weighted degree: it is a global network topology metric that measures the average strength of connections in a weighted network. For a single node, the weighted degree (also called "strength") is the sum of the weights of all edges connected to that node. It reflects the node's importance, considering not only the number of connections (degree) but also the strength or intensity of those connections (e.g., correlation values, interaction scores). (9) Network Diameter (Diameter): the maximum shortest path distance between any two nodes in the network. It measures the network's overall spread. (10) Clustering coefficient: the ratio of the number of actual connections between a node's neighbors to the number of possible connections. It quantifies the local cliquishness or modularity of a node's neighborhood. (11) Centralized betweenness (Betweenness centrality): the frequency with which a node lies on the shortest paths between other nodes. It measures a node's control over information flow and its role as a "bridge" in the network. (12) Natural Connectivity: a robustness metric based on the eigenvalue spectrum of the network's adjacency matrix. It measures the network's stability and resilience to node removal.

Table S5. Effects of different soil amendments on bacterial alpha diversity

	Chao1	Observed_features	Dominance	Goods_coverage	Simpson	Shannon
CK1.RS	1241.906	1212	0.003	0.995	0.997	9.239
CK2.RS	1074.795	1045	0.023	0.995	0.977	8.025
CK3.RS	1103.008	1067	0.043	0.994	0.957	7.585
CK4.RS	953.118	947	0.004	0.998	0.996	8.948
A11.RS	795.773	791	0.004	0.999	0.996	8.698
A12.RS	948.857	940	0.004	0.998	0.996	8.946
A13.RS	1225.982	1179	0.004	0.994	0.996	9.061
A14.RS	1091.619	1066	0.003	0.996	0.997	9.063
A21.RS	991.5	986	0.004	0.998	0.996	8.96
A22.RS	1150.039	1109	0.003	0.995	0.997	9.079
A23.RS	1133.782	1112	0.003	0.995	0.997	9.134
A24.RS	1004.75	995	0.003	0.998	0.997	9.044
A31.RS	1086.47	1078	0.003	0.998	0.997	9.186
A32.RS	1225.619	1193	0.006	0.994	0.994	9.016
A33.RS	1187.04	1150	0.003	0.995	0.997	9.146
A34.RS	1049.25	1037	0.003	0.997	0.997	9.122
A41.RS	1027.784	1018	0.003	0.997	0.997	9.058
A42.RS	1165.614	1126	0.003	0.995	0.997	9.167
A43.RS	1040.562	1020	0.004	0.996	0.996	8.989
A44.RS	945.517	939	0.004	0.998	0.996	8.914
A51.RS	1059.55	1054	0.003	0.998	0.997	9.082
A52.RS	1167.421	1104	0.004	0.993	0.996	8.954
A53.RS	1090.591	1079	0.004	0.997	0.996	8.994
A54.RS	1114.02	1088	0.004	0.996	0.996	9.083
A61.RS	972.94	963	0.004	0.998	0.996	8.967
A62.RS	1144.897	1100	0.003	0.994	0.997	9.048
A63.RS	955.818	937	0.004	0.997	0.996	8.911
A64.RS	1133.515	1099	0.003	0.995	0.997	9.095
A71.RS	994.303	988	0.004	0.998	0.996	8.976
A72.RS	950.558	922	0.004	0.997	0.996	8.819
A73.RS	1061.85	1038	0.003	0.997	0.997	9.092
A74.RS	1059.25	1033	0.004	0.996	0.996	9.028
A81.RS	1017.5	1000	0.005	0.997	0.995	8.812
A82.RS	983	953	0.005	0.996	0.995	8.808
A83.RS	1238.396	1213	0.003	0.995	0.997	9.263
A84.RS	927.03	924	0.004	0.998	0.996	8.833
A91.RS	975.312	965	0.004	0.997	0.996	8.852
A92.RS	1217.571	1169	0.026	0.993	0.974	8.219
A93.RS	1175.895	1128	0.004	0.994	0.996	9.075
A94.RS	1011.958	994	0.003	0.997	0.997	8.995
A101.RS	1060.074	1023	0.004	0.995	0.996	8.95
A102.RS	1009.616	982	0.005	0.996	0.995	8.746

A103.RS	973.083	966	0.004	0.998	0.996	8.838
A104.RS	1193.281	1162	0.003	0.995	0.997	9.226
A111.RS	1011.968	1006	0.004	0.998	0.996	9.035
A112.RS	1036.154	999	0.004	0.996	0.996	8.872
A113.RS	1225.472	1185	0.003	0.994	0.997	9.213
A114.RS	1071.475	1034	0.004	0.996	0.996	8.977
A121.RS	987.725	983	0.003	0.998	0.997	9.034
A122.RS	1150.875	1119	0.003	0.995	0.997	9.118
A123.RS	1144.288	1101	0.003	0.995	0.997	9.098
A124.RS	941.673	932	0.005	0.997	0.995	8.721
A131.RS	966.068	951	0.004	0.997	0.996	8.894
A132.RS	855.833	852	0.005	0.999	0.995	8.648
A133.RS	837.556	832	0.006	0.998	0.994	8.63
A134.RS	965.744	958	0.004	0.998	0.996	8.972
A141.RS	1256.224	1222	0.003	0.994	0.997	9.291
A142.RS	975.044	954	0.004	0.997	0.996	8.85
A143.RS	1018.854	1009	0.003	0.997	0.997	9.064
A144.RS	961.016	954	0.003	0.998	0.997	9.047
A151.RS	1275.79	1222	0.006	0.993	0.994	8.898
A152.RS	1126.267	1094	0.005	0.995	0.995	8.956
A153.RS	982.019	947	0.004	0.996	0.996	8.848
A154.RS	1060.667	1054	0.004	0.997	0.996	9.044

Table S6. Effects of different soil amendments on fungal alpha diversity

	Chao1	Observed_features	Dominance	Goods_coverage	Simpson	Shannon
CK1.RS	972.546	923	0.031	0.997	0.969	6.691
CK2.RS	661.122	646	0.033	0.999	0.967	6.706
CK3.RS	606.5	602	0.028	1	0.972	6.692
CK4.RS	677.879	630	0.08	0.998	0.92	5.156
A11.RS	632.788	624	0.039	0.999	0.961	6.241
A12.RS	717.52	712	0.028	0.999	0.972	6.731
A13.RS	584.957	582	0.016	1	0.984	7.038
A14.RS	647.857	645	0.024	1	0.976	6.789
A21.RS	492.8	479	0.074	0.999	0.926	5.166
A22.RS	694.368	667	0.029	0.998	0.971	6.438
A23.RS	901.988	880	0.02	0.999	0.98	7.259
A24.RS	828.767	822	0.014	0.999	0.986	7.431
A31.RS	827.542	819	0.026	0.999	0.974	7.144
A32.RS	863.694	847	0.023	0.999	0.977	7.046
A33.RS	578.026	557	0.025	0.999	0.975	6.959
A34.RS	809.881	802	0.026	0.999	0.974	7.007
A41.RS	822.542	814	0.016	0.999	0.984	7.207
A42.RS	745.26	733	0.029	0.999	0.971	6.746
A43.RS	985.842	945	0.021	0.998	0.979	7.2
A44.RS	790.646	781	0.023	0.999	0.977	6.863
A51.RS	843.697	800	0.03	0.998	0.97	6.719
A52.RS	811.016	798	0.021	0.999	0.979	7.122
A53.RS	851.25	840	0.018	0.999	0.982	7.255
A54.RS	821.493	811	0.013	0.999	0.987	7.511
A61.RS	960.123	905	0.047	0.998	0.953	6.592
A62.RS	842.222	775	0.032	0.997	0.968	6.426
A63.RS	650.949	642	0.063	0.999	0.937	6.203
A64.RS	744.559	732	0.021	0.999	0.979	6.935
A71.RS	852.094	785	0.092	0.997	0.908	5.997
A72.RS	933.388	926	0.026	0.999	0.974	7.206
A73.RS	886.385	837	0.033	0.997	0.967	6.621
A74.RS	879.644	868	0.02	0.999	0.98	7.179
A81.RS	1017.159	948	0.021	0.997	0.979	7.297
A82.RS	728.368	678	0.035	0.998	0.965	6.33
A83.RS	746.652	737	0.02	0.999	0.98	7.06
A84.RS	945.226	935	0.024	0.999	0.976	7.209
A91.RS	873.103	820	0.026	0.998	0.974	6.87
A92.RS	778.103	734	0.026	0.998	0.974	6.488
A93.RS	751.652	744	0.021	0.999	0.979	6.97
A94.RS	759.479	752	0.022	0.999	0.978	6.945
A101.RS	734	734	0.017	1	0.983	7.234
A102.RS	883.154	855	0.014	0.999	0.986	7.47

A103.RS	987.667	951	0.032	0.998	0.968	7.008
A104.RS	1123.545	1074	0.017	0.997	0.983	7.51
A111.RS	982.642	968	0.015	0.998	0.985	7.485
A112.RS	800.764	791	0.025	0.999	0.975	6.848
A113.RS	918.03	871	0.023	0.998	0.977	6.997
A114.RS	772.549	761	0.02	0.999	0.98	6.956
A121.RS	514.288	511	0.026	1	0.974	6.337
A122.RS	964.221	905	0.02	0.998	0.98	7.187
A123.RS	1017.298	1003	0.015	0.998	0.985	7.671
A124.RS	1085.47	1037	0.013	0.997	0.987	7.653
A131.RS	720.929	707	0.023	0.999	0.977	6.91
A132.RS	1027.784	1009	0.01	0.999	0.99	7.862
A133.RS	1088.094	1047	0.018	0.997	0.982	7.504
A134.RS	965.643	941	0.015	0.998	0.985	7.495
A141.RS	585.628	581	0.092	0.999	0.908	5.541
A142.RS	971.083	906	0.028	0.997	0.972	6.877
A143.RS	980.639	932	0.035	0.998	0.965	6.753
A144.RS	934.684	921	0.02	0.999	0.98	7.284
A151.RS	814.25	800	0.073	0.999	0.927	5.806
A152.RS	1025.609	973	0.035	0.997	0.965	6.594
A153.RS	767.029	753	0.074	0.999	0.926	5.899
A154.RS	719.479	707	0.064	0.999	0.936	5.856

Table S7. Effects of different soil amendments on archaeal alpha diversity

	Chao1	Observed_features	Dominance	Goods_coverage	Simpson	Shannon
CK1.RS	2	2	0.66	1	0.34	0.755
CK2.RS	4	4	0.524	0.957	0.476	1.306
CK3.RS	3	3	0.539	1	0.461	1.149
CK4.RS	4	4	0.403	1	0.597	1.584
A11.RS	2	2	0.773	1	0.227	0.559
A12.RS	5	4	0.633	0.913	0.367	1.053
A13.RS	3	3	0.486	1	0.514	1.28
A14.RS	2	2	0.66	1	0.34	0.755
A21.RS	4	4	0.524	0.957	0.476	1.306
A22.RS	3	3	0.486	1	0.514	1.28
A23.RS	3	3	0.584	1	0.416	1.068
A24.RS	3	3	0.584	1	0.416	1.068
A31.RS	10	8	0.312	0.826	0.688	2.272
A32.RS	6.5	6	0.331	0.913	0.669	2.012
A33.RS	5	5	0.346	0.957	0.654	1.855
A34.RS	6.333	6	0.406	0.913	0.594	1.825
A41.RS	3	3	0.531	1	0.469	1.186
A42.RS	5	4	0.58	0.913	0.42	1.155
A43.RS	3	3	0.531	1	0.469	1.186
A44.RS	3	3	0.501	1	0.499	1.214
A51.RS	3	3	0.637	1	0.363	0.966
A52.RS	6	5	0.463	0.913	0.537	1.562
A53.RS	3	3	0.448	1	0.552	1.353
A54.RS	4	4	0.433	1	0.567	1.527
A61.RS	1	1	1	1	0	0
A62.RS	4	4	0.437	0.957	0.563	1.495
A63.RS	3	3	0.531	1	0.469	1.186
A64.RS	3	3	0.58	1	0.42	1.089
A71.RS	3	3	0.448	1	0.552	1.353
A72.RS	4	4	0.467	1	0.533	1.475
A73.RS	4	4	0.384	1	0.616	1.671
A74.RS	3	3	0.698	1	0.302	0.84
A81.RS	5.5	5	0.429	0.913	0.571	1.614
A82.RS	6.5	6	0.304	0.913	0.696	2.071
A83.RS	3	3	0.486	1	0.514	1.28
A84.RS	3	3	0.595	0.957	0.405	0.998
A91.RS	3	3	0.448	1	0.552	1.353
A92.RS	3	3	0.584	1	0.416	1.068
A93.RS	3	3	0.58	1	0.42	1.089
A94.RS	3	3	0.584	1	0.416	1.068
A101.RS	3	3	0.584	1	0.416	1.068
A102.RS	2	2	0.713	1	0.287	0.667

A103.RS	4	4	0.433	1	0.567	1.527
A104.RS	4	4	0.573	0.957	0.427	1.209
A111.RS	3	3	0.531	1	0.469	1.186
A112.RS	3	3	0.58	1	0.42	1.089
A113.RS	3	3	0.531	1	0.469	1.186
A114.RS	2	2	0.614	1	0.386	0.828
A121.RS	3	3	0.456	1	0.544	1.325
A122.RS	4	4	0.448	0.957	0.552	1.445
A123.RS	3	3	0.539	1	0.461	1.149
A124.RS	3	3	0.486	1	0.514	1.28
A131.RS	3	3	0.58	1	0.42	1.089
A132.RS	3	3	0.584	1	0.416	1.068
A133.RS	3	3	0.38	1	0.62	1.478
A134.RS	3	3	0.584	1	0.416	1.068
A141.RS	3	3	0.49	1	0.51	1.264
A142.RS	4	4	0.437	0.957	0.563	1.495
A143.RS	4	4	0.384	1	0.616	1.671
A144.RS	3	3	0.486	1	0.514	1.28
A151.RS	3	3	0.637	1	0.363	0.966
A152.RS	3	3	0.531	1	0.469	1.186
A153.RS	2	2	0.577	1	0.423	0.887
A154.RS	3	3	0.701	0.957	0.299	0.808

Table S8. Data preprocessing statistics and quality control of rhizosphere soil bacteria

	RawPE	Combined	Qualified	Nochime	Base (nt)	Avglen (nt)	GC (%)	Q20 (%)	Q30 (%)	Effective (%)
CK1.RS	102747	101264	98623	86211	35911181	416.55	57.28	98.60	95.14	83.91
CK2.RS	102955	102023	99515	92397	38190629	413.33	57.15	98.73	95.59	89.75
CK3.RS	102443	101682	99228	92300	38530047	417.44	56.60	98.72	95.36	90.10
CK4.RS	104097	102678	100711	88784	37032058	417.10	57.69	98.62	95.30	85.29
A11.RS	93206	91080	88807	79527	33263719	418.27	57.59	98.58	95.04	85.32
A12.RS	85948	84524	82367	77180	32173242	416.86	57.07	98.65	95.35	89.80
A13.RS	117481	114940	112228	105128	43906041	417.64	57.28	98.41	94.67	89.49
A14.RS	114589	112525	110200	103282	43181747	418.10	57.70	98.48	94.93	90.13
A21.RS	103377	101516	98962	88770	37077675	417.68	57.64	98.67	95.34	85.87
A22.RS	101267	99771	97438	91084	37852268	415.58	57.40	98.74	95.53	89.94
A23.RS	106427	105044	102547	92798	38742929	417.50	57.67	98.65	95.34	87.19
A24.RS	106151	104859	103049	87779	36531272	416.17	57.91	98.67	95.40	82.69
A31.RS	92947	90899	88621	74917	31219705	416.72	57.63	98.63	95.21	80.60
A32.RS	106589	105039	102324	92852	38588121	415.59	56.93	98.75	95.59	87.11
A33.RS	106637	105402	103471	97338	40418789	415.24	57.03	98.67	95.41	91.28
A34.RS	104352	103072	100952	82031	34110766	415.83	57.74	98.64	95.34	78.61
A41.RS	102779	101337	98802	90925	37776913	415.47	57.49	98.64	95.30	88.47
A42.RS	99830	99160	96886	90157	37649884	417.60	57.67	98.73	95.57	90.31
A43.RS	105861	104345	102249	95135	39706444	417.37	57.66	98.66	95.32	89.87
A44.RS	103321	101907	99918	83113	34700222	417.51	58.13	98.58	95.15	80.44
A51.RS	102254	100854	98306	91175	37929015	416.00	57.49	98.58	95.16	89.17
A52.RS	106197	105434	102841	96983	40347014	416.02	57.99	98.71	95.49	91.32
A53.RS	105194	103852	101914	95911	39997022	417.02	57.30	98.62	95.27	91.18
A54.RS	103995	102739	100844	94853	39519119	416.64	57.06	98.67	95.41	91.21

A61.RS	102182	100119	97515	79281	33097184	417.47	58.19	98.61	95.16	77.59
A62.RS	107895	107096	104454	95590	39756428	415.91	57.87	98.70	95.50	88.60
A63.RS	104410	103340	101416	94051	38916074	413.78	57.42	98.70	95.52	90.08
A64.RS	105164	103404	101515	91871	38335907	417.28	56.91	98.63	95.25	87.36
A71.RS	102857	101025	98490	92142	38354158	416.25	57.52	98.70	95.45	89.58
A72.RS	105940	105184	102636	90996	37677421	414.06	57.80	98.72	95.53	85.89
A73.RS	105238	104098	102061	95445	39703377	415.98	57.59	98.61	95.23	90.69
A74.RS	105026	102880	100744	93170	38785332	416.29	57.88	98.50	94.93	88.71
A81.RS	102229	100423	98015	88298	36826347	417.07	57.14	98.68	95.34	86.37
A82.RS	110616	107774	105712	95141	39725790	417.55	57.79	98.65	95.27	86.01
A83.RS	112012	110607	108337	101757	42328818	415.98	57.30	98.75	95.65	90.84
A84.RS	102341	101564	99688	81032	33924995	418.66	57.96	98.69	95.37	79.18
A91.RS	102413	100951	98248	90952	37943563	417.18	57.79	98.63	95.29	88.81
A92.RS	102795	101747	99824	93826	38802938	413.56	56.16	98.75	95.55	91.27
A93.RS	104173	102900	100598	94833	39445951	415.95	57.39	98.71	95.56	91.03
A94.RS	104325	103552	101676	94972	39623806	417.22	57.44	98.69	95.39	91.03
A101.RS	105987	104818	102283	94936	39637451	417.52	57.43	98.74	95.54	89.57
A102.RS	103378	101348	99377	91976	38577581	419.43	57.38	98.66	95.33	88.97
A103.RS	105460	104513	102328	86936	36327070	417.86	57.67	98.82	95.82	82.44
A104.RS	106587	104726	102721	94904	39599881	417.26	57.21	98.67	95.37	89.04
A111.RS	104660	103295	100800	88239	36784025	416.87	57.39	98.68	95.40	84.31
A112.RS	104731	103910	101986	95086	39633396	416.82	57.64	98.68	95.44	90.79
A113.RS	100390	99714	97813	89612	37394847	417.30	57.64	98.73	95.49	89.26
A114.RS	103341	100890	99052	92594	38711405	418.08	57.25	98.68	95.36	89.60
A121.RS	87111	85712	83320	75123	31380070	417.72	57.44	98.57	95.08	86.24
A122.RS	106679	105907	103890	95925	39986149	416.85	58.10	98.65	95.33	89.92

A123.RS	115703	114719	111961	104345	43558366	417.45	57.33	98.77	95.68	90.18
A124.RS	103695	101416	99499	92582	38715966	418.18	57.26	98.59	95.22	89.28
A131.RS	106435	104551	101934	88199	36950225	418.94	57.46	98.63	95.26	82.87
A132.RS	105099	104253	102115	91633	38371282	418.75	57.77	98.58	95.17	87.19
A133.RS	103460	101645	100030	84992	35493914	417.61	57.95	98.61	95.11	82.15
A134.RS	104389	102000	100103	90396	37719297	417.27	57.54	98.63	95.29	86.60
A141.RS	104085	103425	101215	93257	38630719	414.24	57.69	98.81	95.81	89.60
A142.RS	104885	103294	101301	94071	39348920	418.29	57.76	98.60	95.25	89.69
A143.RS	107534	105699	103536	88304	36664463	415.21	57.58	98.60	95.26	82.12
A144.RS	104544	103247	101447	93408	38763028	414.99	57.44	98.63	95.17	89.35
A151.RS	104159	103418	101331	89651	37303476	416.10	57.86	98.80	95.72	86.07
A152.RS	103603	102177	100265	93007	38808990	417.27	57.95	98.66	95.38	89.77
A153.RS	104744	102434	100437	88525	37032376	418.33	58.09	98.61	95.19	84.52
A154.RS	103834	102817	100837	89676	37533235	418.54	58.06	98.73	95.50	86.36

Note: (1) rawPE indicates the original PE reads from the machine; (2) combined is the sequence of Tags obtained by connection; (3) Qualified is the sequence of Raw Tags after filtering low quality and short length; (4) Nochime is the Tags sequence that is ultimately used for subsequent analysis after filtering the chimera, that is, Effective Tags; (5) The Base is the number of bases in the final Effective Tags; (6) AvgLen is the average length of Effective Tags; Q20 and Q30 are the percentage of bases with base mass values greater than 20 (sequencing error rate less than 1%) and 30 (sequencing error rate less than 0.1%) in Effective Tags. (7) GC (%) represents the content of GC bases in Effective Tags; (8) Effective (%) is the percentage of the number of Nochime to the number of rawPE.

Table S9. Data preprocessing statistics and quality control of rhizosphere soil fungi

	RawPE	Combined	Qualified	Nochime	Base (nt)	Avglen (nt)	GC (%)	Q20 (%)	Q30 (%)	Effective (%)
CK1.RS	102505	77091	76854	74101	17922781	241.87	50.68	99.44	97.55	72.29
CK2.RS	197185	85925	85702	84014	21649993	257.70	52.75	99.19	96.17	42.61
CK3.RS	103103	55621	55188	53978	13812758	255.90	49.00	98.84	96.48	52.35
CK4.RS	194275	74304	74095	71640	18231278	254.48	53.00	99.09	95.16	36.88
A11.RS	107821	69816	69671	60506	14464245	239.05	53.12	99.35	96.83	56.12
A12.RS	76916	54523	54384	48673	11665476	239.67	52.28	99.31	96.87	63.28
A13.RS	103185	72035	71911	60219	14312065	237.67	52.64	99.43	97.33	58.36
A14.RS	102646	53629	53447	46663	11390183	244.09	52.38	98.74	95.10	45.46
A21.RS	106438	76371	76240	65389	15270749	233.54	53.71	99.53	97.71	61.43
A22.RS	103469	85051	84903	76816	18266962	237.80	51.56	99.51	97.77	74.24
A23.RS	194358	97061	96812	88558	21895199	247.24	52.03	99.22	96.42	45.56
A24.RS	105797	56659	56498	50150	12276870	244.80	51.51	99.20	96.45	47.40
A31.RS	102668	73412	73247	60847	14339617	235.67	52.75	99.48	97.60	59.27
A32.RS	74055	49571	49462	45949	10865289	236.46	52.21	99.39	97.11	62.05
A33.RS	106764	80578	80344	75495	18592797	246.28	51.18	99.44	97.38	70.71
A34.RS	110104	61122	60941	55309	13561626	245.20	51.87	99.20	96.29	50.23
A41.RS	104112	65522	65371	57769	13833753	239.47	51.25	99.36	97.11	55.49
A42.RS	102126	64406	64252	59321	13992962	235.89	51.52	99.50	97.76	58.09
A43.RS	204300	93247	92997	87803	21268782	242.23	50.58	99.27	96.66	42.98
A44.RS	104481	71726	71536	62492	15038260	240.64	51.55	99.37	97.24	59.81
A51.RS	109644	72654	72487	66294	16097792	242.82	51.27	99.43	97.39	60.46
A52.RS	104695	56444	56278	47906	11638569	242.95	51.48	99.40	97.38	45.76
A53.RS	103057	71364	71206	62255	15157066	243.47	51.36	99.31	96.80	60.41
A54.RS	110587	51911	51695	47204	11466162	242.91	50.52	99.15	96.64	42.68

A61.RS	199978	76630	76456	71802	17724790	246.86	52.14	99.11	95.63	35.90
A62.RS	197291	94370	94184	85120	20505325	240.90	52.93	99.22	96.27	43.14
A63.RS	112986	56409	56205	49996	12276000	245.54	53.06	99.06	96.33	44.25
A64.RS	106138	59140	58986	53343	12650919	237.16	49.59	99.41	97.40	50.26
A71.RS	103526	83659	83527	74730	16764200	224.33	49.88	99.63	98.29	72.18
A72.RS	103192	69597	69424	64185	15542620	242.15	51.14	99.38	97.23	62.20
A73.RS	106115	87592	87385	80355	19693728	245.08	50.61	99.50	97.81	75.72
A74.RS	107032	66095	65918	57470	13758960	239.41	50.82	99.38	97.18	53.69
A81.RS	115873	86258	86011	71257	17768295	249.36	52.04	99.36	97.23	61.50
A82.RS	105971	83841	83670	74544	18048411	242.12	52.84	99.34	97.21	70.34
A83.RS	102670	58004	57859	53223	12866481	241.75	50.86	99.36	97.15	51.84
A84.RS	105606	68213	68013	63028	15031361	238.49	49.83	99.23	96.48	59.68
A91.RS	103136	79545	79364	71319	16920752	237.25	52.14	99.48	97.60	69.15
A92.RS	104612	90755	90305	87426	22822903	261.05	48.82	99.23	97.12	83.57
A93.RS	106625	54756	54598	51052	12612253	247.05	50.93	99.34	97.14	47.88
A94.RS	103211	72502	72288	63111	14975435	237.29	51.28	99.47	97.68	61.15
A101.RS	110564	50705	50580	44246	10974988	248.04	51.58	99.25	96.61	40.02
A102.RS	115772	79370	79181	70064	17002116	242.67	51.96	99.38	97.22	60.52
A103.RS	195055	85281	85049	78930	19545044	247.63	51.55	99.13	95.69	40.47
A104.RS	194551	83336	83073	76347	18223696	238.70	50.89	99.29	96.87	39.24
A111.RS	105784	72616	72432	63967	15671570	244.99	51.45	99.38	97.25	60.47
A112.RS	103753	70839	70708	62558	15117613	241.66	52.79	99.41	97.32	60.30
A113.RS	105412	80756	80557	71480	17185239	240.42	51.61	99.37	97.29	67.81
A114.RS	103430	63267	63086	55041	13070872	237.48	52.47	99.30	96.81	53.22
A121.RS	103853	53408	53315	45684	10920177	239.04	52.27	99.44	97.30	43.99
A122.RS	114745	79896	79658	71258	17322651	243.10	50.78	99.38	97.31	62.10

A123.RS	104864	67150	66954	62337	15089003	242.06	49.74	99.44	97.64	59.45
A124.RS	196876	81292	81026	73140	17950991	245.43	50.31	99.33	97.17	37.15
A131.RS	102998	61579	61442	54417	13065531	240.10	52.78	99.41	97.30	52.83
A132.RS	105721	56118	55907	53234	13151935	247.06	49.37	99.33	97.18	50.35
A133.RS	197834	82411	82079	77936	19873642	255.00	50.06	99.13	96.24	39.39
A134.RS	106646	62210	62001	56869	14179939	249.34	50.54	99.34	97.11	53.33
A141.RS	103040	65398	65289	60642	14011441	231.05	50.33	99.59	98.09	58.85
A142.RS	110574	85043	84836	77953	18672404	239.53	51.07	99.49	97.76	70.50
A143.RS	194765	85927	85706	80109	19594365	244.60	51.52	99.22	96.21	41.13
A144.RS	105169	64876	64207	57638	15059060	261.27	51.46	97.95	94.42	54.81
A151.RS	103770	62900	62724	60366	14943897	247.55	52.76	99.28	96.66	58.17
A152.RS	195660	101321	101006	97673	25005754	256.02	48.61	99.25	96.55	49.92
A153.RS	196253	62470	62304	60587	15112975	249.44	50.59	98.98	94.93	30.87
A154.RS	102394	66208	66066	59302	13967053	235.52	52.23	99.40	97.07	57.92

Note: (1) rawPE indicates the original PE reads from the machine; (2) combined is the sequence of Tags obtained by connection; (3) Qualified is the sequence of Raw Tags after filtering low quality and short length; (4) Nochime is the Tags sequence that is ultimately used for subsequent analysis after filtering the chimera, that is, Effective Tags; (5) The Base is the number of bases in the final Effective Tags; (6) AvgLen is the average length of Effective Tags; Q20 and Q30 are the percentage of bases with base mass values greater than 20 (sequencing error rate less than 1%) and 30 (sequencing error rate less than 0.1%) in Effective Tags. (7) GC (%) represents the content of GC bases in Effective Tags; (8) Effective (%) is the percentage of the number of Nochime to the number of rawPE.

Table S10. Effects of different soil amendments on leaf traits

Treatment	Plant height (cm)	Stem diameter (mm)	Leaf SPAD	Leaf thickness (mm)	Leaf area (cm^2)	Spike length (cm)	Specific leaf area ($cm^2 \cdot g^{-1}$)	Specific leaf weight ($g \cdot cm^{-2}$)	Leaf tissue density ($g \cdot cm^{-3}$)
CK	116.00±3.19bc	5.66±0.09ef	56.45±1.00abc	0.190±0.004cd	74.73±4.47de	17.38±0.43ab	357.69±3.87bc	0.0037±0.0001abcd	0.026±0.003a
A1	105.42±1.61e	5.07±0.39f	43.90±0.86e	0.168±0.003e	61.58±2.94g	14.29±0.18f	335.62±28.48bc	0.0031±0.0002e	0.018±0.001c
A2	117.25±1.44abc	7.42±0.41a	53.65±0.97c	0.193±0.008bcd	77.35±1.68bcde	16.13±0.72bcde	375.53±18.12abc	0.0039±0.0002abc	0.023±0.002ab
A3	110.25±0.85de	6.82±0.37abcd	49.25±0.79d	0.188±0.005cd	78.13±1.59bcde	14.88±0.43ef	383.71±7.37ab	0.0036±0.0001abcde	0.021±0.001bc
A4	112.75±1.03cd	5.91±0.13e	54.70±1.42bc	0.183±0.003d	73.38±0.94ef	15.25±0.25def	392.30±13.20bc	0.0032±0.0001de	0.019±0.001c
A5	111.75±0.63cd	6.51±0.29bcde	56.00±1.64abc	0.188±0.003cd	78.95±1.49abcde	15.75±0.66cde	354.84±12.85c	0.0035±0.0001abcde	0.020±0.000bc
A6	119.25±1.11ab	7.22±0.17ab	59.10±1.20ab	0.208±0.005a	82.70±3.29abcd	18.50±0.20a	332.19±6.86bc	0.0040±0.0003a	0.020±0.001bc
A7	114.25±1.80bcd	6.36±0.20cde	56.65±1.51abc	0.200±0.0001abc	79.13±0.61abcde	16.13±0.13bcde	369.24±12.68bc	0.0037±0.0003abcd	0.021±0.002bc
A8	116.25±3.35abc	5.67±0.29ef	59.58±2.01a	0.205±0.009ab	67.23±1.68fg	16.50±0.29bcd	356.20±25.92bc	0.0040±0.0001ab	0.022±0.001bc
A9	113.75±2.78bcd	6.03±0.12de	52.95±0.73cd	0.180±0.0001de	76.23±2.74cde	15.38±0.24def	369.18±19.03bc	0.0037±0.0001abcd	0.022±0.0001bc
A10	115.25±0.25bcd	6.22±0.13cde	56.50±1.76abc	0.190±0.006cd	81.35±1.96abcde	16.50±0.29bcd	358.39±14.26bc	0.0037±0.0001abcd	0.020±0.001bc
A11	116.50±0.50abc	6.51±0.08bcde	59.90±1.79a	0.193±0.003bcd	84.80±3.01ab	17.00±0.41bc	359.35±8.48bc	0.0034±0.00001bcde	0.019±0.0001bc
A12	110.00±0.58de	7.02±0.51abc	54.15±1.81c	0.183±0.003d	78.08±3.15bcde	14.25±0.25f	373.78±11.45abc	0.0037±0.0003abcd	0.022±0.002bc
A13	115.50±0.50bcd	7.33±0.17ab	52.55±0.58cd	0.198±0.005abc	84.10±1.79abc	15.75±0.48cde	366.06±13.44bc	0.0035±0.0002abcde	0.019±0.001c
A14	113.00±0.71cd	6.77±0.15abcd	59.08±1.68ab	0.193±0.003bcd	86.95±2.92a	15.50±0.29def	425.87±33.10a	0.0033±0.00001de	0.019±0.0001c
A15	121.50±2.25a	5.77±0.17ef	56.60±0.97abc	0.200±0.0001abc	82.23±2.55abcd	17.00±0.68bc	391.80±6.11ab	0.0033±0.00001cde	0.018±0.0001c

Note: CK, control group; A1, Continuous cropping obstacle soil (CCOS); A2, CCOS + biochar; A3, CCOS + earthworm castings; A4, CCOS + sodium bentonite; A5, CCOS + fly ash; A6, CCOS + humic acid; A7, CCOS + desulfurization gypsum; A8, CCOS + calcium-magnesium-phosphate fertilizer; A9, CCOS + phosphogypsum; A10, CCOS + *Bacillus subtilis*; A11, CCOS + *Bacillus megatherium*; A12, CCOS + seaweed fertilizer; A13, CCOS + *Bacillus mucilaginosus*; A14, Foxtail millet and *Vigna radiata* rotation; A15, Foxtail millet and *Sorghum bicolor* rotation. Different lowercase letters (a, b, c, d, e, f, and g) indicate that the different soil amendments have significant differences (Duncan's test, $p < 0.05$).

Table S11. Effects of different soil amendments on root traits

Treatment	Root length (cm)	Root surface area (cm^2)	Average root diameter (mm)	Root volume (cm^3)	Root tips	Root forks	Specific root surface area ($cm^2 \cdot g^{-1}$)	Root tissue density ($g \cdot cm^{-3}$)
CK	53.53±3.40ab	52.41±2.84abc	3.18±0.29a	4.23±0.58ab	97.00±17.72abc	151.00±19.75ab	0.15±0.03cd	2.06±0.57bc
A1	28.70±5.97b	38.73±0.72c	4.91±1.02a	4.76±0.98ab	46.00±18.97c	54.25±11.33b	0.29±0.03a	2.79±0.70abc
A2	39.82±6.66ab	41.43±2.88bc	3.62±0.65a	3.75±0.70ab	78.50±13.97abc	94.50±21.23ab	0.25±0.06abc	3.09±0.87abc
A3	40.46±10.09ab	54.34±5.05ab	4.95±0.92a	6.45±0.78a	58.50±16.99bc	91.00±23.31ab	0.20±0.01abcd	1.84±0.35c
A4	46.86±12.30ab	49.82±6.67abc	4.21±1.15a	5.25±1.54ab	77.00±24.47abc	131.50±38.27ab	0.20±0.04abcd	2.46±0.87abc
A5	50.52±14.47ab	51.40±7.04abc	3.60±0.46a	4.45±0.48ab	83.50±20.05abc	151.25±70.22ab	0.15±0.01bcd	1.80±0.36c
A6	41.28±12.52ab	51.93±6.63abc	4.90±1.30a	6.21±1.68a	64.75±15.43bc	113.75±43.25ab	0.21±0.04abcd	2.03±0.60bc
A7	44.77±7.06ab	44.52±1.73abc	3.41±0.53a	3.84±0.70ab	89.50±21.53abc	112.50±22.56ab	0.22±0.03abcd	2.81±0.53abc
A8	46.58±13.16ab	43.05±4.11abc	3.76±1.11a	4.08±1.42ab	89.75±33.45abc	113.50±26.93ab	0.26±0.04abc	3.35±0.82abc
A9	64.87±6.54a	44.85±1.75abc	2.25±0.16a	2.51±0.14b	133.00±20.73a	172.75±18.50a	0.23±0.03abcd	4.23±0.89ab
A10	50.99±10.30ab	49.59±3.59abc	3.46±0.67a	4.24±0.73ab	116.50±24.51ab	136.75±24.98ab	0.24±0.05abcd	2.94±0.61abc
A11	37.82±10.02ab	42.06±4.34bc	4.36±1.18a	4.34±0.87ab	64.75±24.80bc	99.50±33.50ab	0.26±0.04ab	2.89±0.77abc
A12	52.83±6.45ab	41.93±2.95bc	2.69±0.48a	2.86±0.60b	98.50±14.85abc	128.75±20.34ab	0.28±0.04a	4.61±1.06a
A13	43.07±12.32ab	45.15±3.25abc	4.88±1.43a	5.04±1.08ab	67.33±18.47abc	128.00±39.83ab	0.24±0.02abcd	2.41±0.39abc
A14	55.15±8.17ab	52.30±2.08abc	3.14±0.28a	4.07±0.28ab	89.50±8.35abc	147.50±28.38ab	0.18±0.02abcd	2.44±0.54abc
A15	67.90±7.07a	56.45±4.93a	2.67±0.13a	3.77±0.40ab	108.50±4.70abc	198.25±15.07a	0.13±0.01d	2.00±0.26bc

Note: CK, control group; A1, Continuous cropping obstacle soil (CCOS); A2, CCOS + biochar; A3, CCOS + earthworm castings; A4, CCOS + sodium bentonite; A5, CCOS + fly ash; A6, CCOS + humic acid; A7, CCOS + desulfurization gypsum; A8, CCOS + calcium-magnesium-phosphate fertilizer; A9, CCOS + phosphogypsum; A10, CCOS + *Bacillus subtilis*; A11, CCOS + *Bacillus megatherium*; A12, CCOS + seaweed fertilizer; A13, CCOS + *Bacillus mucilaginosus*; A14, Foxtail millet and *Vigna radiata* rotation; A15, Foxtail millet and *Sorghum bicolor* rotation. Different lowercase letters (a, b, c, and d) indicate that the different soil amendments have significant differences (Duncan's test, $p < 0.05$).

Table S12. Effects of different soil amendments on leaf photosynthetic pigment content, crop biomass, and yield

Treatment	Grains diameter (cm)	Leaf weight (g)	Stem weight (g)	Spike weight (g)	Aboveground biomass (g)	Underground biomass (g)	Total biomass (g)	Chlorophyll a	Chlorophyll b	Total chlorophyll	Carotenoids
CK	18.80±0.88bc	13.41±1.80b	26.58±2.73b	47.97±8.49ab	87.96±12.79b	6.82±1.24b	94.78±13.99b	19.77±3.56ab	10.03±3.09a	29.80±6.59a	4.14±0.68a
A1	14.11±0.85d	15.21±0.93ab	28.79±1.37ab	50.65±2.74ab	94.64±4.57ab	11.22±1.40ab	105.86±5.77ab	16.96±1.18b	5.44±0.41a	22.40±1.55a	3.63±0.13a
A2	18.68±0.36bc	17.43±1.14ab	32.78±1.83ab	60.96±2.45ab	111.16±4.92ab	10.09±1.72ab	121.25±5.85ab	21.35±2.60ab	10.54±2.58a	31.89±5.01a	4.36±0.47a
A3	19.54±0.81bc	16.70±1.17ab	32.35±1.13ab	57.59±1.03ab	106.63±2.82ab	11.07±1.04ab	117.71±2.18ab	21.30±1.96ab	10.16±2.92a	31.46±4.56a	3.98±0.34a
A4	19.72±0.64bc	15.66±1.41ab	29.79±1.86ab	58.25±4.06ab	103.70±6.45ab	9.00±1.13ab	112.70±7.07ab	22.46±1.35ab	8.95±1.52a	31.42±2.85a	4.13±0.12a
A5	18.79±0.34bc	14.27±2.53ab	26.48±3.21b	53.83±5.72ab	94.58±11.23ab	7.85±1.48ab	102.43±12.17ab	21.68±1.97ab	8.76±1.75a	30.44±3.67a	3.98±0.24a
A6	22.84±0.68a	19.69±0.87a	36.54±0.62a	64.85±3.00a	121.08±3.83a	10.45±1.57ab	131.52±5.32a	23.79±0.83ab	12.01±2.55a	35.80±3.25a	3.80±0.16a
A7	18.37±0.32c	16.44±1.39ab	29.65±0.57ab	53.83±2.69ab	99.91±3.90ab	9.81±1.18ab	109.72±5.08ab	22.25±1.34ab	12.60±4.43a	34.85±5.10a	3.16±1.06a
A8	18.71±0.98bc	14.66±1.55ab	29.14±2.11ab	44.64±4.09b	88.43±7.62b	10.48±0.62ab	98.92±7.40ab	21.45±2.16ab	9.08±2.28a	30.53±4.41a	4.10±0.20a
A9	18.03±0.31c	16.43±2.20ab	29.89±3.53ab	56.16±7.13ab	102.48±12.48ab	10.25±1.48ab	112.73±13.91ab	19.05±2.55ab	6.30±1.16a	25.35±3.70a	3.81±0.51a
A10	19.70±0.92bc	17.44±2.34ab	32.50±3.93ab	63.20±8.06a	113.14±14.27ab	12.12±2.60a	125.26±16.74ab	17.72±2.69ab	7.42±1.51a	25.14±3.65a	3.17±0.44a
A11	20.58±0.45b	18.73±1.19ab	33.10±2.92ab	61.55±5.34ab	113.38±8.93ab	10.79±1.31ab	124.17±10.24ab	18.25±2.02ab	6.05±1.29a	24.30±3.31a	3.53±0.23a
A12	18.38±0.27c	16.41±1.48ab	30.23±2.18ab	55.07±2.85ab	101.70±5.98ab	11.51±1.07ab	113.21±7.04ab	24.19±0.49a	10.82±1.13a	35.01±1.59a	4.54±0.33a
A13	19.98±0.06bc	16.37±1.85ab	31.02±2.75ab	60.85±6.09ab	108.23±10.62ab	7.84±2.70ab	116.08±11.61ab	24.18±0.68a	11.63±1.95a	35.81±2.54a	4.28±0.14a
A14	18.17±0.25c	17.80±2.00ab	31.87±2.22ab	58.59±7.14ab	108.26±10.76ab	9.50±1.37ab	117.75±11.74ab	21.17±2.98ab	10.63±3.00a	31.80±5.83a	3.65±0.37a
A15	19.20±0.63bc	16.27±1.70ab	29.40±1.65ab	54.07±6.09ab	99.73±9.07ab	7.24±0.54ab	106.97±8.98ab	22.45±0.81ab	8.23±0.62a	30.68±1.43a	4.18±0.10a

Note: CK, control group; A1, Continuous cropping obstacle soil (CCOS); A2, CCOS + biochar; A3, CCOS + earthworm castings; A4, CCOS + sodium bentonite; A5, CCOS + fly ash; A6, CCOS + humic acid; A7, CCOS + desulfurization gypsum; A8, CCOS + calcium-magnesium-phosphate fertilizer; A9, CCOS + phosphogypsum; A10, CCOS + *Bacillus subtilis*; A11, CCOS + *Bacillus megatherium*; A12, CCOS + seaweed fertilizer; A13, CCOS + *Bacillus mucilaginosus*; A14, Foxtail millet and *Vigna radiata* rotation; A15, Foxtail millet and *Sorghum bicolor* rotation. Different lowercase letters (a, b, c, and d) indicate that the different soil amendments have significant differences (Duncan's test, $p < 0.05$).

Table S13. Effects of different soil amendments on nutritional quality of leaves and fruits

Treatment	Leaf total starch ($mg \cdot g^{-1}$)	Leaf soluble sugar ($mg \cdot g^{-1}$)	Fruit total starch ($mg \cdot g^{-1}$)	Fruit soluble starch ($mg \cdot g^{-1}$)	Fruit soluble sugar ($mg \cdot g^{-1}$)	Fruit ether extract ($mg \cdot g^{-1}$)	Fruit crude protein ($mg \cdot g^{-1}$)
CK	16.68±0.54a	25.41±1.70ab	65.63±8.02bc	28.20±3.59a	8.11±0.41a	30.38±4.44a	86.92±9.43a
A1	16.34±1.17a	29.05±1.13ab	68.18±5.14bc	25.94±8.50a	8.49±0.33a	30.83±2.85a	84.23±8.55a
A2	16.63±1.15a	24.61±1.70b	52.89±4.98bc	23.07±1.53a	7.91±0.25a	32.16±2.25a	95.61±3.00a
A3	16.59±0.75a	29.65±3.23ab	58.03±3.44bc	27.31±4.28a	7.98±0.36a	28.60±2.62a	89.52±3.30a
A4	17.25±2.06a	26.12±2.90ab	66.25±10.90bc	19.99±0.96a	7.68±0.20a	30.78±3.19a	94.45±3.97a
A5	16.07±1.08a	24.91±1.22b	64.09±9.07bc	19.99±2.49a	8.26±0.55a	33.43±5.06a	97.16±2.83a
A6	15.28±0.42a	27.92±2.00ab	56.63±9.39bc	18.32±5.40a	7.23±0.25a	36.68±4.30a	108.03±5.95a
A7	18.11±1.85a	32.50±2.15a	98.40±26.78a	20.39±2.97a	8.52±1.06a	38.10±2.30a	87.93±3.78a
A8	17.62±1.94a	26.69±2.39ab	71.01±4.20abc	23.30±5.32a	8.09±0.58a	30.87±2.22a	99.12±3.13a
A9	16.18±0.46a	27.63±0.54ab	78.04±9.26ab	18.39±1.75a	7.91±0.40a	30.71±5.17a	103.59±21.43a
A10	15.35±1.13a	27.61±4.57ab	72.08±8.52abc	20.91±4.94a	8.00±0.62a	37.20±1.69a	105.54±2.64a
A11	17.23±1.43a	26.15±1.59ab	51.65±5.50bc	24.18±7.40a	7.20±0.25a	32.18±4.92a	101.83±6.98a
A12	15.86±0.82a	22.79±1.36b	55.18±4.51bc	20.60±5.44a	8.07±0.48a	29.40±4.57a	92.02±2.91a
A13	19.64±5.14a	24.30±2.76b	53.71±8.04bc	13.48±2.17a	6.88±0.14a	29.34±4.72a	95.40±3.43a
A14	18.07±2.23a	27.31±0.88ab	45.00±2.49c	16.76±1.37a	7.93±0.73a	34.82±2.95a	82.80±8.48a
A15	17.60±0.66a	28.36±1.67ab	52.57±3.14bc	17.56±0.59a	7.71±0.29a	36.21±4.67a	86.32±8.78a

Note: CK, control group; A1, Continuous cropping obstacle soil (CCOS); A2, CCOS + biochar; A3, CCOS + earthworm castings; A4, CCOS + sodium bentonite; A5, CCOS + fly ash; A6, CCOS + humic acid; A7, CCOS + desulfurization gypsum; A8, CCOS + calcium-magnesium-phosphate fertilizer; A9, CCOS + phosphogypsum; A10, CCOS + *Bacillus subtilis*; A11, CCOS + *Bacillus megatherium*; A12, CCOS + seaweed fertilizer; A13, CCOS + *Bacillus mucilaginosus*; A14, Foxtail millet and *Vigna radiata* rotation; A15, Foxtail millet and *Sorghum bicolor* rotation. Different lowercase letters (a, b, and c) indicate that the different soil amendments have significant differences (Duncan's test, $p < 0.05$).

Table S14. Effects of different soil amendments on leaf physicochemical properties

Treatment	Leaf pH	Leaf EC ($\mu S \cdot cm^{-1}$)	Leaf OC ($g \cdot kg^{-1}$)	Leaf TN ($g \cdot kg^{-1}$)	Leaf TP ($g \cdot kg^{-1}$)	Leaf TK ($g \cdot kg^{-1}$)
CK	5.20±0.10a	296.00±62.18a	473.66±3.30abc	15.41±1.01ab	1.78±0.23ab	16.36±0.82a
A1	5.40±0.11a	178.75±55.12a	475.84±7.07abc	16.66±2.76ab	1.42±0.14abc	15.91±1.02a
A2	5.38±0.09a	391.25±111.36a	463.73±9.90abc	13.44±0.29b	1.27±0.07c	15.36±0.73a
A3	5.44±0.16a	275.75±85.32a	480.70±8.69abc	14.41±0.38ab	1.49±0.07abc	16.72±0.60a
A4	5.34±0.19a	351.00±85.32a	473.88±5.84abc	16.14±1.11ab	1.58±0.16abc	15.80±0.56a
A5	5.41±0.23a	369.50±79.69a	477.74±6.23abc	15.83±0.65ab	1.54±0.11abc	17.56±0.87a
A6	5.43±0.25a	443.00±105.09a	473.69±1.11abc	18.20±1.08a	1.90±0.12a	17.72±1.34a
A7	5.45±0.15a	467.25±164.37a	465.13±4.58abc	14.20±0.79b	1.54±0.21abc	16.24±1.17a
A8	5.33±0.10a	252.50±97.66a	456.44±13.21c	15.57±0.91ab	1.73±0.16abc	16.71±0.88a
A9	5.21±0.26a	244.50±93.32a	470.48±9.09abc	13.31±1.44b	1.48±0.26abc	14.61±1.54a
A10	5.35±0.12a	323.50±36.14a	488.08±6.20a	15.98±0.58ab	1.62±0.02abc	16.97±0.89a
A11	5.36±0.16a	402.75±122.52a	485.40±1.35ab	14.65±1.00ab	1.41±0.10abc	16.81±1.12a
A12	5.16±0.22a	397.00±93.53a	481.16±15.54abc	14.16±0.66b	1.40±0.11abc	15.88±0.52a
A13	5.26±0.17a	288.25±59.60a	481.31±2.51abc	14.78±1.04ab	1.34±0.16bc	15.77±0.54a
A14	5.23±0.16a	332.25±98.43a	476.44±7.98abc	14.02±0.98b	1.30±0.12bc	15.50±0.98a
A15	5.32±0.11a	319.50±62.02a	460.21±8.82bc	16.05±1.17ab	1.73±0.12abc	16.83±0.81a

Note: CK, control group; A1, Continuous cropping obstacle soil (CCOS); A2, CCOS + biochar; A3, CCOS + earthworm castings; A4, CCOS + sodium bentonite; A5, CCOS + fly ash; A6, CCOS + humic acid; A7, CCOS + desulfurization gypsum; A8, CCOS + calcium-magnesium-phosphate fertilizer; A9, CCOS + phosphogypsum; A10, CCOS + *Bacillus subtilis*; A11, CCOS + *Bacillus megatherium*; A12, CCOS + seaweed fertilizer; A13, CCOS + *Bacillus mucilaginosus*; A14, Foxtail millet and *Vigna radiata* rotation; A15, Foxtail millet and *Sorghum bicolor* rotation. Different lowercase letters (a, b, and c) indicate that the different soil amendments have significant differences (Duncan's test, $p < 0.05$).

Table S15. Effects of different soil amendments on root nutrients

Treatment	Root OC ($g\cdot kg^{-1}$)	Root TN ($g\cdot kg^{-1}$)	Root TP ($g\cdot kg^{-1}$)	Root TK ($g\cdot kg^{-1}$)
CK	433.93±7.91ab	8.09±0.62a	0.95±0.13ab	10.62±0.48abcd
A1	444.77±11.47ab	7.89±1.15a	0.68±0.04b	10.59±0.49abcd
A2	423.82±4.97ab	7.35±0.22a	0.69±0.05b	10.66±0.37abcd
A3	447.96±11.80ab	7.40±0.22a	0.74±0.05ab	9.79±0.42bcd
A4	433.68±6.38ab	7.69±0.17a	0.84±0.07ab	8.90±1.12d
A5	451.85±12.85a	7.50±0.55a	0.74±0.03ab	10.36±1.09abcd
A6	435.62±16.87ab	8.50±0.87a	0.80±0.04ab	12.65±0.75a
A7	445.92±6.31ab	9.26±1.48a	0.91±0.13ab	11.01±0.89abcd
A8	415.30±3.11b	8.52±0.33a	1.02±0.08a	12.06±0.80ab
A9	446.56±5.11ab	7.71±0.30a	0.90±0.08ab	11.53±0.41abc
A10	436.75±14.61ab	8.00±0.56a	0.79±0.05ab	9.26±0.73cd
A11	448.29±12.63ab	7.74±0.22a	0.76±0.05ab	11.13±0.63abcd
A12	443.82±9.31ab	7.62±0.34a	0.95±0.17ab	10.35±0.45abcd
A13	427.07±17.30ab	7.68±0.38a	0.99±0.20ab	10.30±0.66abcd
A14	433.66±6.42ab	8.03±0.25a	0.88±0.04ab	11.37±0.55abc
A15	434.50±3.16ab	7.49±0.41a	0.83±0.05ab	12.34±0.75a

Note: CK, control group; A1, Continuous cropping obstacle soil (CCOS); A2, CCOS + biochar; A3, CCOS + earthworm castings; A4, CCOS + sodium bentonite; A5, CCOS + fly ash; A6, CCOS + humic acid; A7, CCOS + desulfurization gypsum; A8, CCOS + calcium-magnesium-phosphate fertilizer; A9, CCOS + phosphogypsum; A10, CCOS + *Bacillus subtilis*; A11, CCOS + *Bacillus megatherium*; A12, CCOS + seaweed fertilizer; A13, CCOS + *Bacillus mucilaginosus*; A14, Foxtail millet and *Vigna radiata* rotation; A15, Foxtail millet and *Sorghum bicolor* rotation. Different lowercase letters (a, b, c, and d) indicate that the different soil amendments have significant differences (Duncan's test, $p < 0.05$).

Table S16. Effects of various soil amendments on the soil physicochemical properties

Treatment	Soil pH	Soil EC ($\mu S \cdot cm^{-1}$)	Soil OC ($g \cdot kg^{-1}$)	Soil TN ($g \cdot kg^{-1}$)	Soil TP ($g \cdot kg^{-1}$)	Soil TK ($g \cdot kg^{-1}$)	Soil AN ($mg \cdot kg^{-1}$)	Soil AP ($mg \cdot kg^{-1}$)	Soil AK ($mg \cdot kg^{-1}$)
CK	8.16±0.16a	100.00±5.48c	15.59±0.64f	0.62±0.01d	0.85±0.02b	22.32±0.21ab	24.58±1.15c	71.99±6.63cd	137.00±3.34f
A1	8.03±0.03ab	113.50±5.68c	23.18±0.56bcd	1.00±0.01abc	1.06±0.14ab	23.16±0.55ab	35.56±1.58ab	96.66±9.42bcd	282.00±8.81ab
A2	7.90±0.09bc	115.50±3.95c	32.36±3.71a	1.05±0.05ab	1.10±0.16ab	23.47±0.41ab	29.63±5.85bc	98.05±14.95bcd	301.00±16.94a
A3	7.89±0.06bc	116.50±3.50c	26.30±0.72b	1.12±0.01a	1.06±0.14ab	23.77±0.42ab	39.39±1.35a	85.39±10.84bcd	287.75±9.90ab
A4	7.89±0.05bc	121.00±2.08c	19.17±2.04cdef	0.98±0.01bc	1.10±0.17ab	22.93±0.47ab	35.21±3.87ab	206.81±58.89a	267.50±12.01ab
A5	7.89±0.05bc	108.00±5.60c	20.91±2.39cde	0.99±0.03bc	1.13±0.16ab	23.07±0.15ab	34.16±1.03ab	168.38±34.43ab	258.00±18.14abc
A6	7.85±0.05bc	120.50±4.27c	19.78±0.77cdef	1.03±0.03ab	1.14±0.17ab	23.86±0.78a	38.17±2.57a	137.88±35.94abc	302.00±13.06a
A7	6.83±0.05d	2311.00±140.68a	24.33±0.99bc	0.99±0.07bc	1.64±0.45a	22.01±0.43b	33.29±2.17ab	124.46±30.60abcd	198.75±6.75e
A8	8.16±0.01a	149.00±3.42c	20.67±1.25cdef	0.95±0.04bc	1.12±0.17ab	23.04±1.32ab	35.91±0.92ab	114.80±36.29bcd	269.75±6.73ab
A9	6.81±0.06d	1932.00±67.68b	20.54±1.33cdef	0.97±0.02bc	1.11±0.15ab	22.70±0.31ab	34.16±2.15ab	57.88±13.19cd	218.75±19.14cde
A10	7.84±0.02bc	131.50±10.05c	20.47±1.41cdef	1.04±0.03ab	1.04±0.17ab	23.56±0.79ab	36.08±1.72ab	52.07±12.63cd	246.50±18.72bcd
A11	7.80±0.04c	120.50±2.50c	23.51±0.18bcd	1.01±0.02abc	1.12±0.18ab	23.17±0.33ab	36.43±1.72ab	43.40±15.80d	263.50±5.81ab
A12	7.89±0.05bc	113.00±7.19c	21.20±0.74cde	0.92±0.08bc	1.12±0.20ab	22.92±0.24ab	35.21±1.94ab	59.85±23.03cd	247.50±19.94bcd
A13	7.92±0.05bc	126.00±4.08c	18.69±0.35def	0.89±0.03c	1.10±0.20ab	22.44±0.30ab	33.64±0.96ab	79.94±23.32bcd	247.75±12.77bcd
A14	7.89±0.08bc	111.00±4.04c	22.32±1.85bcd	0.93±0.06bc	1.21±0.19ab	23.50±0.14ab	33.47±1.24ab	78.40±21.86cd	214.25±17.89de
A15	8.00±0.09abc	113.50±7.04c	16.82±1.62ef	0.71±0.01d	0.76±0.01b	23.13±0.35ab	25.80±1.03c	82.06±26.73bcd	152.50±5.56f

Note: CK, control group; A1, Continuous cropping obstacle soil (CCOS); A2, CCOS + biochar; A3, CCOS + earthworm castings; A4, CCOS + sodium bentonite; A5, CCOS + fly ash; A6, CCOS + humic acid; A7, CCOS + desulfurization gypsum; A8, CCOS + calcium-magnesium-phosphate fertilizer; A9, CCOS + phosphogypsum; A10, CCOS + *Bacillus subtilis*; A11, CCOS + *Bacillus megatherium*; A12, CCOS + seaweed fertilizer; A13, CCOS + *Bacillus mucilaginosus*; A14, Foxtail millet and *Vigna radiata* rotation; A15, Foxtail millet and *Sorghum bicolor* rotation. Different lowercase letters (a, b, c, d, e, and f) indicate that the different soil amendments have significant differences (Duncan's test, $p < 0.05$).

Figure S1

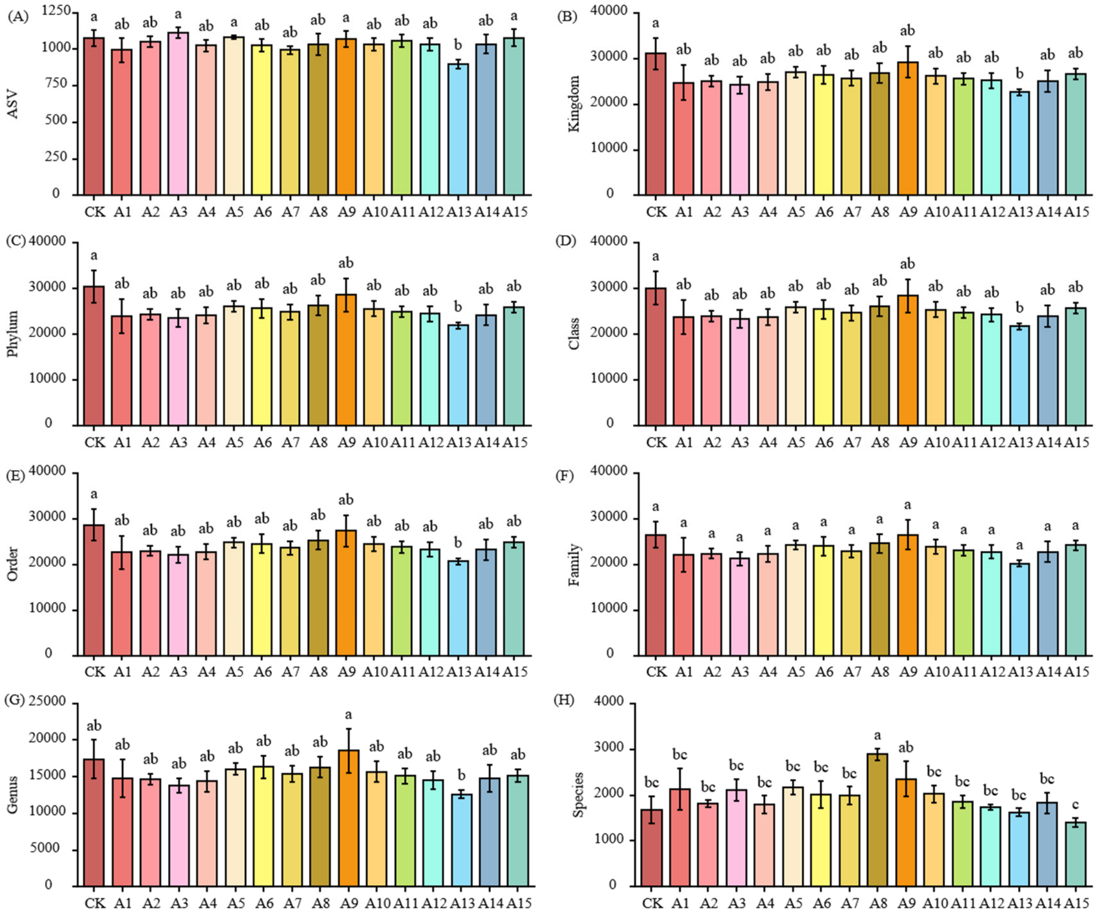

Figure S1. Effects of different soil amendments on the number of bacterial ASVs, Kingdom, Phylum, Class, Order, Family, Genus, and Species

Note: CK, control group; A1, Continuous cropping obstacle soil (CCOS); A2, CCOS + biochar; A3, CCOS + earthworm castings; A4, CCOS + sodium bentonite; A5, CCOS + fly ash; A6, CCOS + humic acid; A7, CCOS + desulfurization gypsum; A8, CCOS + calcium-magnesium-phosphate fertilizer; A9, CCOS + phosphogypsum; A10, CCOS + *Bacillus subtilis*; A11, CCOS + *Bacillus megatherium*; A12, CCOS + seaweed fertilizer; A13, CCOS + *Bacillus mucilaginosus*; A14, Foxtail millet and *Vigna radiata* rotation; A15, Foxtail millet and *Sorghum bicolor* rotation. Different lowercase letters (a, b, and c) indicate that the different soil amendments have significant differences (Duncan's test, $p < 0.05$).

Figure S2

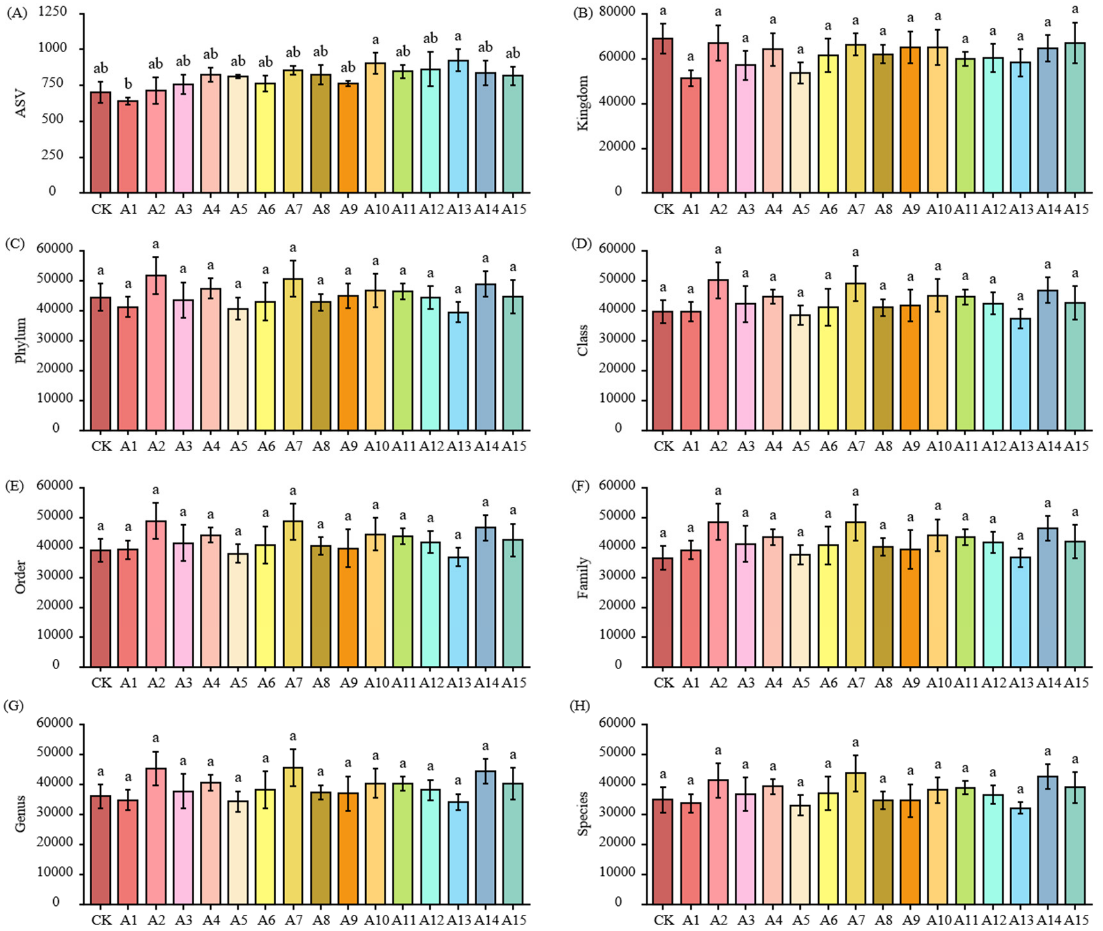

Figure S2. Effects of different soil amendments on the number of fungal ASVs, Kingdom, Phylum, Class, Order, Family, Genus, and Species

Note: CK, control group; A1, Continuous cropping obstacle soil (CCOS); A2, CCOS + biochar; A3, CCOS + earthworm castings; A4, CCOS + sodium bentonite; A5, CCOS + fly ash; A6, CCOS + humic acid; A7, CCOS + desulfurization gypsum; A8, CCOS + calcium-magnesium-phosphate fertilizer; A9, CCOS + phosphogypsum; A10, CCOS + *Bacillus subtilis*; A11, CCOS + *Bacillus megatherium*; A12, CCOS + seaweed fertilizer; A13, CCOS + *Bacillus mucilaginosus*; A14, Foxtail millet and *Vigna radiata* rotation; A15, Foxtail millet and *Sorghum bicolor* rotation. Different lowercase letters (a and b) indicate that the different soil amendments have significant differences (Duncan's test, $p < 0.05$).

Figure S3

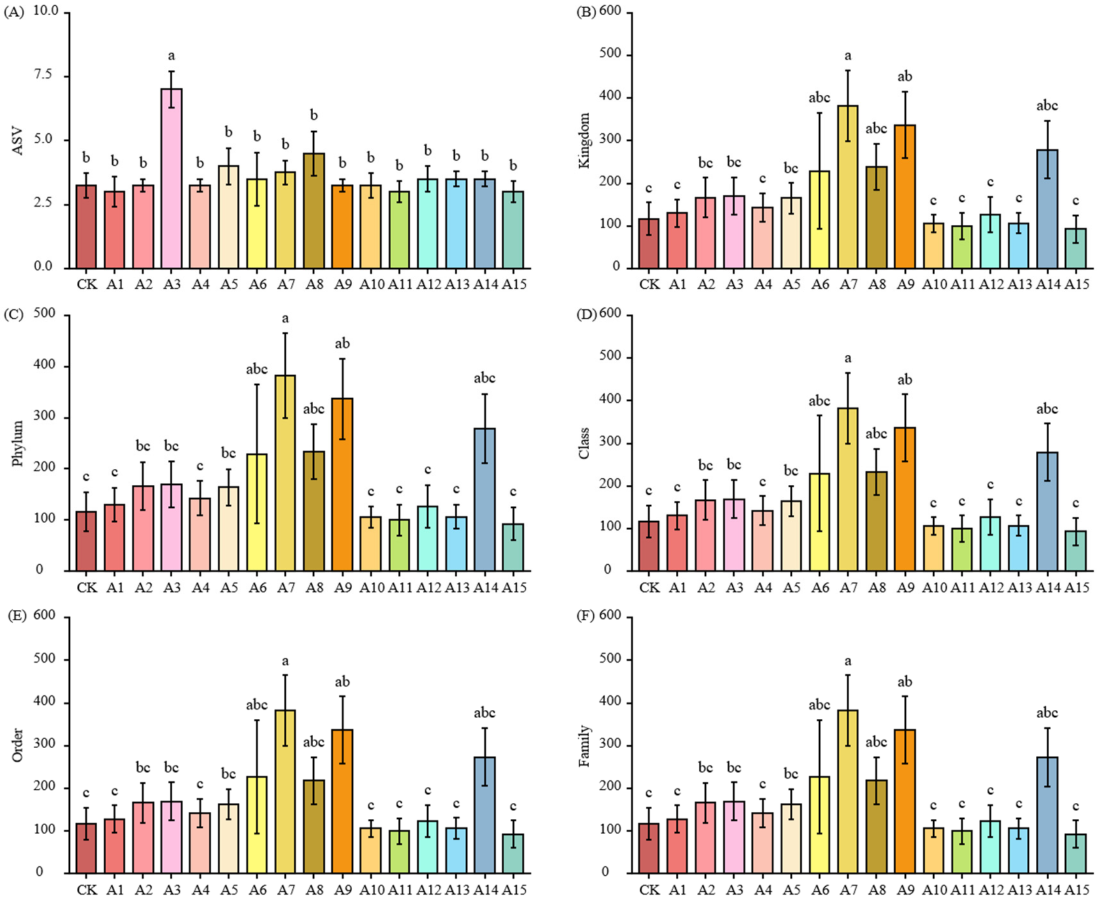

Figure S3. Effects of different soil amendments on the number of archaeal ASVs, Kingdom, Phylum, Class, Order, Family, Genus, and Species

Note: CK, control group; A1, Continuous cropping obstacle soil (CCOS); A2, CCOS + biochar; A3, CCOS + earthworm castings; A4, CCOS + sodium bentonite; A5, CCOS + fly ash; A6, CCOS + humic acid; A7, CCOS + desulfurization gypsum; A8, CCOS + calcium-magnesium-phosphate fertilizer; A9, CCOS + phosphogypsum; A10, CCOS + *Bacillus subtilis*; A11, CCOS + *Bacillus megatherium*; A12, CCOS + seaweed fertilizer; A13, CCOS + *Bacillus mucilaginosus*; A14, Foxtail millet and *Vigna radiata* rotation; A15, Foxtail millet and *Sorghum bicolor* rotation. Different lowercase letters (a, b, and c) indicate that the different soil amendments have significant differences (Duncan's test, $p < 0.05$).
